# Extremely stretchable and self-healing conductor based on thermoplastic elastomer for all-three-dimensional printed triboelectric nanogenerator

**DOI:** 10.1038/s41467-019-10061-y

**Published:** 2019-05-14

**Authors:** Kaushik Parida, Gurunathan Thangavel, Guofa Cai, Xinran Zhou, Sangbaek Park, Jiaqing Xiong, Pooi See Lee

**Affiliations:** 1 School of Materials Science and Engineering, Nanyang Technological University, 50 Nanyang Avenue, Singapore, 639798 Singapore; 2 Singapore-HUJ Alliance for Research and Enterprise (SHARE), Nanomaterials for Energy and Water Nexus (NEW), Campus for Research Excellence and Technological Enterprise (CREATE), 1 Create Way, Singapore, 138602 Singapore

**Keywords:** Devices for energy harvesting, Electronic devices

## Abstract

Advances in next-generation soft electronic devices rely on the development of highly deformable, healable, and printable energy generators to power these electronics. Development of deformable or wearable energy generators that can simultaneously attain extreme stretchability with superior healability remains a daunting challenge. We address this issue by developing a highly conductive, extremely stretchable, and healable composite based on thermoplastic elastomer with liquid metal and silver flakes as the stretchable conductor for triboelectric nanogenerators. The elastomer is used both as the matrix for the conductor and as the triboelectric layer. The nanogenerator showed a stretchability of 2500% and it recovered its energy-harvesting performance after extreme mechanical damage, due to the supramolecular hydrogen bonding of the thermoplastic elastomer. The composite of the thermoplastic elastomer, liquid metal particles, and silver flakes exhibited an initial conductivity of 6250 S cm^−1^ and recovered 96.0% of its conductivity after healing.

## Abstract (web summary)

Development of wearable devices relies on advance in power sources with complementary mechanical properties. Here the authors use a conductive, stretchable and healable composite to achieve sustained performance in a triboelectric nanogenerator under extreme deformation and after severe mechanical damage.

## Introduction

The rapid growth of soft electronic devices has propelled the need for highly deformable electronic devices, including transistors^1,2^, sensors^3–7^, energy-storage devices^8–10^, and light-emitting diodes (LEDs)^11,12^. Advances and practical utilization of these soft-electronic devices rely on the realization of a mechanically durable, stretchable, and healable power source to drive these devices. Among the various energy harvesters, the triboelectric nanogenerators (TENGs) reported by Wang and colleagues^13,14^ have emerged as a promising power source for self-powered devices because of their ability to harvest energy from ambient mechanical motions such as movements of the human body, which makes them suitable for wearable and soft-electronic devices. In addition, TENGs are characterized by high output voltage, high power density, high energy-conversion efficiency, environmental friendliness, and low fabrication costs^15–19^.

Extensive efforts have been made to fabricate deformable and healable TENGs by adopting various approaches. For example, structural designs such as serpentine-patterned electrodes^20^, interlocking kirigami^21^, and three-dimensional (3D) network structures have been developed^21^; however, the stretchability obtained with these approaches is limited to 22%, 100%, and 310%, respectively. Embedding conducting fillers (such as silver (Ag) nanowires^22^, Ag nanosheets^21^, Ag nanofibers^23^, carbon nanotubes^23^, carbon grease^23^, carbon black^24^, and liquid metal^25^) in a stretchable matrix (such as polydimethylsiloxane (PDMS) and silicone rubber) can significantly improve the stretchability. However, the stretchability is limited to 700% owing to the elastic limit of the elastomeric matrices. The use of ionic conductors such as hydrogels results in high stretchability of 1000%^26–28^; however, hydrogels are limited by long-term instability and low mechanical toughness. Despite these efforts, the maximum stretchability of TENGs is limited to ~1000%. Moreover, most of the stretchable triboelectric layers are based on commercial elastomers such as PDMS, silicone rubber, and VHB tape^22^. Thus, there is a need to develop triboelectric materials and conductors with superior stretchability and mechanical properties. A few attempts have been made to fabricate healable TENGs from self-healing vitrimer elastomers^29^, dynamic amine bonds with PDMS^30^, and polyurethane-based shape memory polymer^31^. However, stretchability of these self-healing nanogenerators are unsatisfactory. Simultaneously, attaining extreme stretchability (with good energy-harvesting performance) and healability (completely restoring its performance after mechanical damage) is still a daunting challenge^32^.

For the realization of a deformable and mechanically durable nanogenerator, one of the key challenges is the interface compatibility of the triboelectric layer and the conductor. Due to the Young’s modulus mismatch, cyclic stretching leads to the delamination of the two layers, resulting in the degradation of performance. Herein, we address this problem by developing a stretchable and healable TENG (SH-TENG) with high stretchability and good healability enabled by the use of a stretchable and healable polyurethane acrylate (PUA) elastomer as the triboelectric layer and also as the polymer matrix for the conductor (consisting of liquid metal, silver flakes, and PUA). The extreme stretchability can be attributed to the supramolecular hydrogen bonding of the PUA matrix that is used as both the triboelectric layer and the current collector matrix. The dynamic multivalent H-bonding of the designed supramolecular PUA reversibly breaks up and reforms to support the desired stretchability and healability. Liquid metal and silver flakes were embedded in the PUA matrix to perform as the conductive fillers in which the liquid metal provided the electrical connection between the silver flakes to maintain the electrical conductivity during extreme stretchability. To the best of our knowledge, the obtained stretchability of 2500% is the highest compared with other reported stretchable TENGs. The nanogenerator sustained its energy-harvesting performance under extreme deformation and after severe mechanical damage. To the best of our knowledge, this is the first report of an all-3D-printed TENG, and that with the highest stretchability. Practical applicability of the energy harvester is demonstrated by using it to power LEDs. The resulting SH-TENG showed record-high stretchability with healability and 3D printability, thus pushing the boundaries of deformable energy harvesters.

## Results

### Materials characterizations

**Fig. 1:**
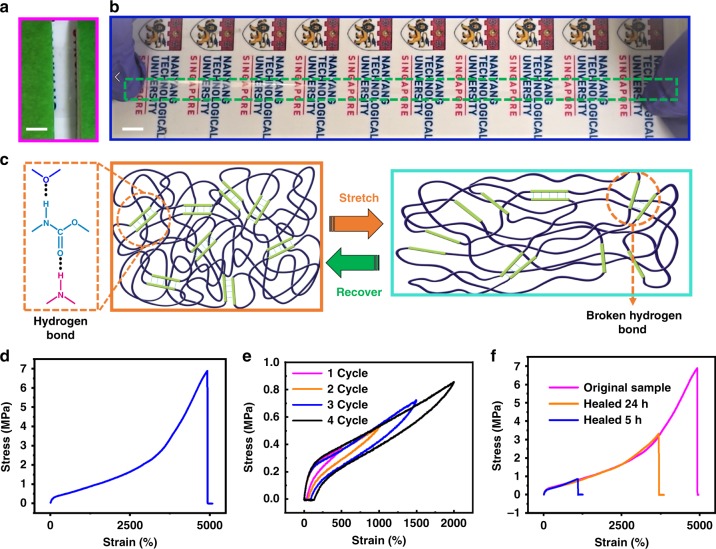
Digital photo and mechanical properties of the stretchable polyurethane acrylate elastomer. Digital photo of the PUA elastomer at strains of **a** 0% and **b** 3000%. Scale bar: 5 mm. **c** Schematic illustration of the breaking and reforming of hydrogen bonding resulting in high stretchability. **d** Tensile stress–strain behavior of the polyurethane acrylate (PUA) film. **e** Tensile loading and unloading data of the PUA for evaluation of its hysteresis behavior. **f** Stress–strain behavior of the self-healed PUA film with different healing time at 100 °C. Deformation rate for all measurements: 100 mm min^−1^

Figure 1a, b and Supplementary Fig. 1 shows the digital photos of the developed transparent, 3D printable, highly stretchable, and healable PUA-based thermoplastic elastomer. The presence of characteristic peaks of the polyurethane prepolymer (NCO-terminated prepolymer, PU–NCO) and the PUA based on 2-hydroxyethyl methacrylate (PUA–HEMA) in the nuclear magnetic resonance (NMR) and fourier transform infrared (FTIR) spectra confirms the successful synthesis of PUA (Supplementary Figs. 2 and 3). Detailed descriptions of the chemical structures, Raman spectra, and ultraviolet–visible (UV–vis) spectra are provided in Supplementary Figs. 4, 5, 6, 7, and 8. PUA consists of rubbery soft segments (SSs) and rigid hard segments (HSs)^33^. Owing to the distinct thermodynamic incompatibility between the SSs and HSs, microphase separation occurred in the segmented PUA and a distinct domain structure was formed^34^. The different segments resulted in two glass-transition temperatures (*T*_g_)—low *T*_g_ for the SS and high *T*_g_ for the HS—which are reflected in the differential scanning calorimetry (DSC) and dynamic mechanical analysis (DMA) results (Supplementary Figs. 9 and 11). The thermal stability of the PUA is shown and explained in Supplementary Fig. 10. The binary hard–soft domains of PUA were prepared from isophoronediisocyanate (IPDI) and HEMA. The functionality of the hydroxyl groups in HEMA and isocyanates (NCO groups) in IPDI could be increased to form crosslinked polymers. The supramolecular H-bonding was verified by the presence of peaks at 3333 and 3365 cm^−1^ (H-bonding between N–H groups, Supplementary Fig. 3d) and the peaks at 1720 and 1729 cm^−1^ (H-bonding formed between C=O groups, Supplementary Fig. 3c). The crosslinking density was tuned by incorporating HEMA into PU. The addition of a higher amount of HEMA led to a chain-extension reaction, resulting in further crosslinking, which is reflected by the increase in intensity of the FTIR peaks at 3333 and 3365 cm^−1^ (Supplementary Fig. 3d). The tunable physical properties of the thermoplastic polyurethane were directly related to their degree of crosslinking, in which the HS-rich regions act as thermally reversible crosslinks that control the stiffness and elasticity of the elastomers, while the SS enhances the toughness and facilitates self-healing^34,35^.

### Mechanical properties of PUA

The mechanical properties of PUA were evaluated with different concentrations of HEMA (hereafter denoted as PUA–*X*%HEMA, where *X*% is the weight ratio of HEMA). Increasing the HEMA content leads to superior mechanical properties with improved stretchability, fracture toughness, and tensile strength (Fig. 1d and Supplementary Fig. 12a). Compared with the other PUA–HEMA samples, PUA–20%HEMA shows superior mechanical properties with a stretchability of 5000% (Supplementary Movie 1). Supplementary Table 3 shows a comparison of mechanical properties with previously reported works on acrylate-based self-healing polymers. Compared with the various acrylate-based self-healing polymers, there exists a double crosslinked network and non-covalent physical crosslinking network in our synthesized PUA. Under relaxed (0% strain) conditions, the inter-domain space formed via the large number of H-bonds resulted in the folding of segmented polymer chains. Stretching leads to preferential breaking of dynamic H-bonds and dissipates the bond energy, resulting in the unfolding of segmented polymer chains (schematically illustrated in Fig. 1c)^36^. Thus, extreme stretchability can be attributed to the presence of supramolecular hydrogen bonding. Increasing the HEMA content to 30 wt% lowers the stretchability and fracture energy of PUA–30%HEMA owing to the reduction in both the crosslinking density and the distribution of the hard domains^37^. The hysteresis observed in tensile loading–unloading cyclic test of the PUA–20%HEMA indicates the dissipation of energy due to the breakage of hydrogen bonds (Fig. 1e). The optimum PUA–20%HEMA (hereafter PUA–20%HEMA is referred as PUA) was selected for self-healing evaluation. Polarized optical micrograph shows the healing of the bifurcated PUA film (Supplementary Fig. 13). We found that the PUA demonstrated good healability with a mechanical healing efficiency of 45.1% after it was subjected to complete bifurcation (Fig. 1f, Supplementary Movie 2). The healability of PUA can be attributed to the breaking and recovery of multiple H-bonds in the supramolecular polymer matrix (schematically illustrated in Fig. 1c). Mechanical damage (complete bifurcation) led to the breakage of intramolecular hydrogen bonds and covalent bonds (type I: [−NH···O=C−]; type II: [−NH···O = C-O-]). The bifurcated PUA was healed at 100 °C for 24 h and subsequently allowed to heal for 24 h at room temperature. Manual connection at room temperature initiated the recovery of H-bonding and higher temperature led to faster recovery of the H-bonding^35^. The mechanical properties of the PUA before and after healing is summarized in Supplementary Table 2.

### Electrical properties of the SH-TENG

**Fig. 2:**
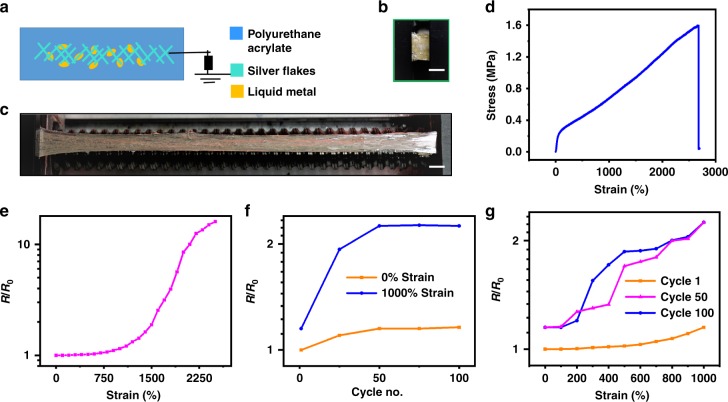
Schematic diagram, digital photo, and mechanical and electrical properties. **a** Schematic diagram of the stretchable and healable triboelectric nanogenerator (SH-TENG). Silver flakes and liquid metal particles are embedded in the polyurethane acrylate (PUA) matrix. Digital photo of the SH-TENG at strains of **b** 0% and **c** 2500%. Scale bar: 5 mm. **d** Tensile stress–strain behavior of the SH-TENG device. Deformation rate: 100 mm min^−1^. **e** Changes in resistance of the SH-TENG device at various uniaxial strains. *R*/*R*_0_ is the relative resistance, where *R*_0_ is the resistance of the conductor without strain and *R* is the resistance of the conductor with a certain strain. **f** Changes in resistance of the SH-TENG device for 100 cycles of uniaxial strain. **g** Changes in resistance of the SH-TENG device at various uniaxial strains measured for 100 cycles

In addressing the interfacial compatibility of stretchable conductor and triboelectric active layer, we adopted PUA–20%HEMA (hereafter PUA–20%HEMA is referred as PUA) as the polymer matrix for the stretchable conductor and as the triboelectric layer. Figure 2a–c show the schematic and digital photo of the constructed SH-TENG. The SH-TENG was prepared by embedding liquid metal particles (eutectic gallium indium particles (EGaInPs)) and metal particles (silver flakes) in the PUA film. Liquid metal particles (EGaInPs particles with a thin shell layer of Ga_2_O_3_) are distributed throughout the polymer matrix and act as electrical anchors between the Ag flakes to provide additional conducting paths^7^. The fabrication process of the SH-TENG is described in Methods section and schematically illustrated in Supplementary Fig. 14. PUA serves as the stretchable and healable matrix, and the liquid metal particles act as the electrical anchors between the silver flakes to maintain the conductivity at extreme stretchability. The amount of PUA, silver flakes, and liquid metal particles used was optimized in the weight ratio of 1:1:2 (Supplementary Fig. 15). The OH groups in the PUA matrix are bonded with the fatty acid present on the surface of the commercial Ag flakes^38^ (Supplementary Fig. 6). The SH-TENG can be stretched up to 2500% (Fig. 2d, Supplementary Movie 3). The stretchable conductor of the SH-TENG shows a high conductivity of 6250 S cm^−1^ with excellent mechanical properties. The fabricated SH-TENG could sustain its high conductivity at a record-high stretchability of 2500% (Fig. 2e–g, data are collected by two terminal measurements across the SH-TENG). Upon stretching, the liquid metal particle shells break and release the conductive liquid metal to provide the required electrical anchor between the separated Ag flakes in the PUA matrix. When the conductor is stretched beyond extreme stretchability of 1500%, the reducing percolation conductive pathways lead to an increase in resistance. The relative resistance, *R*/*R*_0_ (where *R*_0_ is the resistance of the conductor without strain and *R* is the resistance of the conductor with a certain strain) of the conductor at 2500% strain is 10, clearly indicating that the electrical properties are robust under high strain (Fig. 2e). The durability and stability of the conductor were evaluated by measuring the relative resistance change when it was subjected to 100 cycles of repetitive strain of 1000% and stretched at a rate of 100 mm min^−1^ (Fig. 2f, g). Moreover, the conductor was electrically healable: after the sample was healed (heated at 100 °C for 24 h and subsequently allowed to heal for 24 h at room temperature (30 °C), it recovered with an electrical healing efficiency of 96.0% (Supplementary Fig. 16a). Field-emission scanning electron microscopy (FE-SEM) images of the conductor before and after healing shows the recovery of the damaged parts (Supplementary Fig. 17). The recovery of the electrical resistance of the stretchable conductor after five cycles of healing process indicates the repetitive self-healing behavior of the stretchable conductor (Supplementary Fig. 16b).

### Mechanism of the SH-TENG

**Fig. 3:**
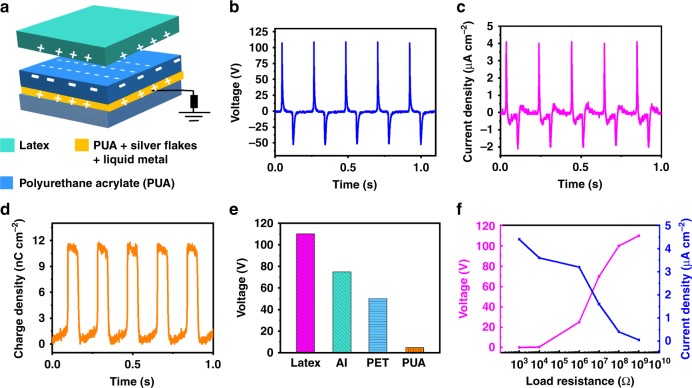
Schematic diagram and energy-harvesting performance. **a** Schematic diagram of the complete stack of stretchable and healable triboelectric nanogenerator (SH-TENG), where latex is used a the positive triboelectric layer, polyurethane acrylate (PUA) is used as the negative triboelectric layer and (polyurethane acrylate + silver flakes + liquid metal) is used as the current collector. The performance of the SH-TENG was evaluated when it was subjected to a mechanical force of 40 N at a frequency of 5 Hz. **b** Output voltage (*V*_op_), **c** current density (*I*_s_), **d** charge-transfer density (*σ*_T_), and **e** output voltage of the SH-TENG with PUA as the negative triboelectric material and different positive materials (latex, aluminum (Al), polyethylene terephthalate (PET), and PUA). **f** Variations in output voltage, current, and power density as functions of external impedance

A schematic of the complete stack of SH-TENG (where latex is used as the positive triboelectric layer and PUA is used as the negative triboelectric layer) is shown in Fig. 3a. To improve the performance of the TENG, microstructures were developed on the PUA layer using sandpaper as a template (Supplementary Fig. 18). The performance of the single-electrode TENG was measured by connecting it to a reference electrode through an external load. The device generated energy upon application of mechanical force owing to the coupled effects of tribo-electrification and electrostatic induction^15,22,39^. The operating mechanism of the SH-TENG is schematically illustrated in Supplementary Fig. 19. Owing to the relatively low electronegativity of PUA, it was used as the triboelectrically negative material and the latex with less electronegativity served as the triboelectrically positive material^22^. Before mechanical force was applied, there was no contact between the two materials (PUA and latex). Therefore, no surface charges were generated on the films. Upon application of mechanical force (40 N at a frequency of 5 Hz), the two layers came into contact with each other. With the difference in their electronegativity, charge transfer from the latex film to PUA, making the latex layer positively charged and the PUA film negatively charged^24^. When the force was released, the two oppositely charged surfaces became separated in space, resulting in a potential difference. As a result of this built-up potential difference, a transient charge flowed from the current collector to the ground, resulting in a current flow. When the separation between the two charged surfaces reached a maximum, electrostatic equilibrium was obtained. The potential difference and the short-circuit charge developed during the separation of the two polymeric films are represented by Supplementary equations 2 and 3, respectively. Upon reapplying of the force, the process was reversed, resulting in a current flow in the opposite direction^16,36^. Repeated application of mechanical force resulted in the generation of multiple output current. The mechanism on the change in the output performance of SH-TENG upon changing the applied deformation is explained in Supplementary Note 12 and Supplementary Fig. 20.

### Energy-harvesting performance of the SH-TENG

The energy-harvesting performance of the SH-TENG was evaluated by measuring the output voltage (*V*_op_), short-circuit current density (*I*_sc_), and charge-transfer density (*σ*_T_) upon the application of compressive mechanical impact. The SH-TENG generates an approximate *V*_op_ of 100 V (Fig. 3b), *I*_sc_ of 4 μA cm^−2^ (Fig. 3c), and *σ*_T_ of 12 nC cm^−2^ (Fig. 3d). To simulate a real working environment, the energy-harvesting performance of the SH-TENG was measured under mechanical impact of different magnitudes (Supplementary Fig. 21). When the magnitude of the mechanical force was increased, the effective contact area between the latex and PUA increased due to the microscopic deformations at the interface, thus enhancing the performance. The energy-harvesting performance of the SH-TENG depends on the thickness of the triboelectric layer (PUA layer) (Supplementary Fig. 22). In real practical applications, the single-electrode TENGs are subjected to mechanical impact from different materials; thus, the output voltages of the SH-TENG are measured by using several materials (such as latex, aluminum, polyethylene terephthalate, and PUA) as the triboelectric positive material (Fig. 3e). The device configuration with latex shows the highest voltage, because it has the largest difference between the positive and negative triboelectric layers in the triboelectric series^22^. As the energy-harvesting performance of a triboelectric device depends on the external load impedance, the performance of the SH-TENG can be obtained across different load resistances ranging from 1 KΩ to 1 GΩ. Following Ohm’s law, when the load resistance increases, the output voltage increases and the output current decreases (Fig. 3f). An output power density of 40 μW cm^−2^ can be obtained across an optimum load resistance of 1 MΩ (Fig. 3f). Finally, the long-term stability of the SH-TENG was evaluated by subjecting the device to continuous mechanical impact. The device shows no obvious degradation in performance after 50,000 continuous cycles of mechanical impact (force: 40 N; frequency: 5 Hz), clearly indicating a mechanically robust and reliable device for practical applications (Supplementary Fig. 23). The mechanical robustness can be attributed to the interface compatibility of the matrix of the conductor and the triboelectric layer, where PUA is used both as the triboelectric layer and as the polymer matrix for the conductor. The developed materials can also be used in lateral sliding mode. The device schematic and the voltage output of the lateral sliding mode is provided in Supplementary Fig. 24).

### Performance of the nanogenerator under extreme deformations

**Fig. 4:**
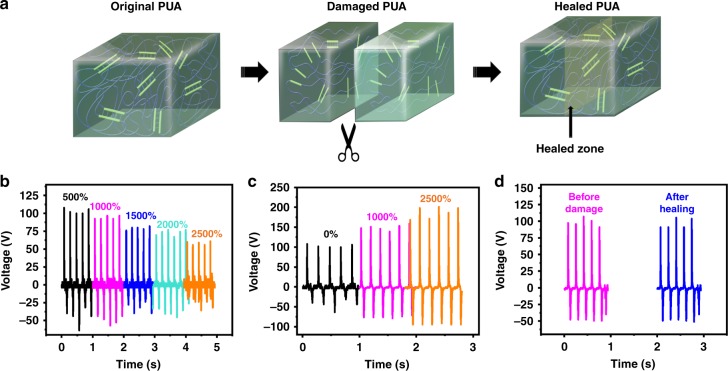
Illustration of self-healing of polyurethane acrylate and energy-harvesting performance. **a** Schematic illustration of the healing of the polyurethane acrylate (PUA) conductor via breaking and reforming of hydrogen bonding. **b** The output voltage of the stretchable and healable triboelectric nanogenerator (SH-TENG) subjected to an impact of 40 N at a frequency of 5 Hz under various uniaxial strains. The measurements were carried out by straining the PUA layer of the SH-TENG device, while the area of the impacting force (area of the top latex layer, 3 × 3 cm^2^) was kept constant. **c** The output voltage of the SH-TENG subjected to an impact of 40 N at a frequency of 5 Hz under various uniaxial strains, when the area of the latex is increased so that it was similar to the uniaxial strain of the device. **d** Output voltage of the SH-TENG before mechanical damage and after healing

To demonstrate that the SH-TENG can be used as an extremely deformable power source for electronic devices, its performance was evaluated by subjecting the device to strenuous mechanical strains from 0 to 2500% (Fig. 4b). The measurements were carried out by straining the PUA layer of the SH-TENG device, while the area of the impacting force (area of the top latex layer, 3 × 3 cm^2^) was kept constant. The output voltage and current density (upon application of a mechanical force of 40 N at a frequency of 5 Hz) decrease with an increase in the uniaxial strain. As the uniaxial strain increased, the microstructures on PUA surface became elastically deformed and the effective contact area exposed to the exerted force decreased (when the impact area is fixed), thus reducing the net surface charge and the energy-harvesting performance. After the SH-TENG was restored to the initial unstrained condition after extreme deformation, its performance was close to that in the original state (before deformation), thus indicating its ability to recover the device performance after strenuous mechanical deformations (Supplementary Fig. 25). When the area of the impacting force (area of the top latex layer) is increased so that it was similar to the uniaxial strain of the device, the output voltage increased accordingly, indicating that extreme stretchability could improve the energy-harvesting performance of TENG (Fig. 4c). This is attributed to the increased surface area of contact between the two triboelectric layer, which dominates the overall performance. The detailed explanation of the change in the output voltage under strain is explained in Supplementary Note 15 and Fig. 26. To evaluate the durability of the SH-TENG, the energy-harvesting performance (when subjected to a mechanical force of 40 N at a frequency of 5 Hz) was evaluated after every 100 cycles of uniaxial stretching up to 1000% strain for a total of 500 cycles (Supplementary Fig. 27). To further evaluate the robustness and healability of the energy harvester, the performance of the SH-TENG was evaluated after subjecting the device to severe mechanical damage, including healability after complete bifurcation (Fig. 4a–d). The energy-harvesting performance (upon subjecting the SH-TENG to a mechanical force of 40 N at a frequency of 5 Hz) of the healed device after bifurcation was almost unchanged, thus indicating its ability to restore itself after severe mechanical damage (Fig. 4d). Voltage output of the SH-TENG before damaged and after self-healing is demonstrated in Supplementary Movie 4. This extremely SH-TENG is a promising candidate as a power source for soft and deformable electronics. To the best of our knowledge, the obtained stretchability is the highest when compared with that of previously reported energy harvesters (a detailed comparison is provided in Supplementary Table 4).

### Three-dimensional printable, stretchable, and healable nanogenerator

**Fig. 5:**
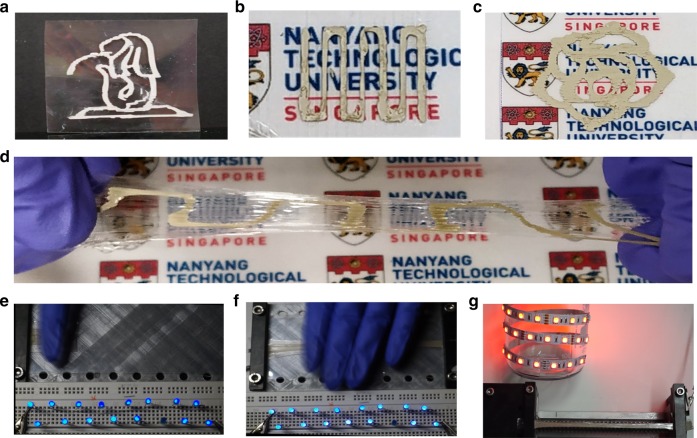
Digital photos of the 3D printable PUA layer and elastic conductor in various shapes. **a** Merlion shape. **b** Serpentine shape. **c** Flower shape. **d** Stretched serpentine pattern. **e** Digital photo of the stretchable and healable triboelectric nanogenerator (SH-TENG) powering light-emitting diodes (LEDs) by finger tapping. **f** Digital photo of the SH-TENG under extreme deformation (2000% axial strain) and powering LEDs by palm tapping. **g** Digital photo demonstrating the high conductivity of the SH-TENG

Incorporation of additive manufacturing enables the fabrication of devices with different geometries, thus ensuring effective integration of energy harvesters with different shapes and sizes of electronics devices. Recently, efforts have been made to develop 3D printable TENG, such as 3D printable chalcogenide TENG^37^, flexible hinges-based TENG^40^, and fused deposition modeling-based 3D printed TENG^41^. These prior works demonstrated the printing of only the triboelectric layer. In addition, most of the commercial 3D printable elastomeric materials such as PDMS (maximum stretchability = 100%) have limited stretchability^42^. Here we report an all-3D printable highly SH-TENG (where both the PUA layer and the electrode were 3D printed). Figure 5a–c shows the digital photo of the all 3D printed SH-TENG (comprising of PUA and (PUA + Ag + lq metal)) printed in various shapes by an extrusion-based 3D printer. The detailed printing process is described in the Methods section. The all 3D printed SH-TENG could generate energy under extreme deformations and mechanical damage, thus making it an ideal energy harvester for portable self-powered electronics. To demonstrate real-time practical applications, the SH-TENG was utilized to power LEDs. Twenty LEDs were powered by tapping the SH-TENG with finger (Fig. 5e and as demonstrated in Supplementary Movie 5). The electrical circuit diagram of the setup is illustrated in Supplementary Fig. 28. Furthermore, the SH-TENG could power LEDs under extreme stretchability (at an axial strain of 2500%), thus demonstrating its ability to act as a patternable and printable power source for deformable electronics (Fig. 5f and as demonstrated in Supplementary Movie 5). In addition, the ability of the SH-TENG to sustain its conductivity at extreme stretchability (2500%) was demonstrated by connecting it in series with a power source and LEDs (Fig. 5g and Supplementary Fig. 29).

## Discussion

We have developed a 3D printable, highly conductive, extremely SH-TENG. The developed nanogenerator shows good conductivity (6250 S cm^−1^), record-high stretchability of 2500%, and healability. The stretchability and healability of the developed PUA can be attributed to the supramolecular hydrogen-bonding interactions with the large number of weak hydrogen bonds, which repeatedly break and reform during mechanical damage. PUA acts as an ultra-stretchable triboelectric layer. In addition to the stretchable PUA matrix, liquid metal provides the electrical anchoring between the silver flakes, thus keeping a stable conductivity at extreme stretchability. Using our the fabricated current collector, the developed nanogenerator shows the highest stretchability when compared with the previously reported nanogenerator devices. In addition, the healable nanogenerator recovers its energy-harvesting performance after mechanical damage. Here we report an all-3D-printable stretchable TENG, where both the electrode and the triboelectric layer is 3D printed. Owing to the combined properties of extreme stretchability, healability, and 3D printability, the developed nanogenerator represents a breakthrough in the domain of deformable energy harvesters.

## Methods

### Materials

IPDI (Sigma-Aldrich, 98%), poly(tetramethylene ether)glycol (PTMG, Sigma-Aldrich, Mn = 1000 gmol^−1^, 98%), 1,1,1-tris(hydroxymethyl)propane (TMP, Sigma-Aldrich, 97%), dibutyltin dilaurate (Sigma-Aldrich, 95%), and HEMA (Sigma-Aldrich, ≥99%), liquid metal (EGaInPs solution, Sigma-Aldrich), silver flakes (Sigma-Aldrich, size = 10 μm, purity > 99%), were purchased from Sigma-Aldrich. Methyl ethyl ketone (Fisher Chemical, ≥99%) was purchased from Fisher Chemical. All analytical grade reagents and solvents were used without further purification.

### Synthesis of the PUA

The UV-curable PUA was synthesized by addition polymerization in two steps, as outlined in the procedures as shown in Supplementary Fig. 4. Prior to the synthesis, PTMG was placed in a dry round-bottomed flask and degassed at 80 °C overnight. A catalyst (0.01 wt %) was then added to the degassed PTMG and the mixture was stirred at 70 °C for 30 min. To synthesize the PU–NCO, IPDI and TMP were reacted at 70 °C for 5 h with the PTMG solution. The NCO/OH mole ratio was fixed at 1:6. The weight fraction (wt %) of NCO in the prepolymer was verified by *n*-butyl amine back titration (ASTM D 2572–97). Then, PU–NCO was end-capped with the required amount of HEMA at 50 °C. This reaction proceeded until the residual NCO content reached the theoretical value. The absence of NCO groups in the final synthesized PUA was verified by FTIR spectroscopy and NMR spectroscopy. To vary the crosslinking density, the HEMA content was varied from 10 to 30 wt%. Finally, the PUA solution was poured onto a glass petri dish and then UV-cured (UV lamp, 80 W, 365 nm) for 1.5 h. The obtained PUA film had a thickness of ~ 200 μm. To increase the surface area of the PUA, microstructures were formed on the surface of PUA by drop casting the PUA solution onto a sandpaper (Grit = P2500, used a template).

### Fabrication of the stretchable and healable nanogenerator

The detailed fabrication process of the stretchable and healable nanogenerator is schematically illustrated in Supplementary Fig. 14. The liquid metal particles were prepared by sonication of the liquid metal in a solution of acetone for 1 h^43,44^. Sonication ensures the formation of stable EGaInPs particles with a thin shell layer of Ga_2_O_3_^45^. Subsequently, it was centrifuged to remove the acetone, leaving behind EGaInPs particles. The conductive slurry was obtained by mixing PUA solution with silver flakes and liquid metal particles in a weight ratio of 1:1:2. The conductive slurry was drop-casted and sandwiched between two PUA films to obtain an embedded structure. Subsequently, the embedded structure was UV-cured for 1.5 h. The embedded conducting layer is connected to the measuring instrument using a copper tape. The dimensions of the device are: device area = 3 × 3 cm^2^, conductor area = 2 × 2 cm^2^.

### Three-dimensional printing of PUA and conductor

The 3D printing of both the PUA and conductor (PUA + silver + liquid metal) was achieved with an extrusion printer System 30 M (Hyrel 3D, USA). The as-prepared PUA was transferred to a syringe and mounted onto the printer. It was uniformly extruded out with a needle of 0.5 mm inner diameter controlled by the printer. After extrusion, the PUA solidified quickly at room temperature. No extra curing time is required with proper control of flow rate. Two layers of PUA were printed to form the PUA film. After printing the PUA film, a single layer of the conductor (PUA + Ag + lq metal) was printed onto the as-printed PUA film. The printing process is the same as that of the PUA film but with a lower printing speed, as it is more viscous.

### Structural characterization and electrical measurement

Structural characterization of the synthesized PUA solution was investigated using ^1^H NMR (400 MHz Bruker DPX 400). The NMR spectra were performed at room temperature using tetramethylsilane as the internal standard in deuterated dimethyl sulfoxide. The FTIR (FTIR-ATR, Perkin Elmer, Frontier) spectroscopy was used to measure the FTIR spectra by scanning each film for 32 times with a resolution of 4 cm^−1^. FE-SEM (JSM 7600 F) was used to observe the morphology of the materials. The thickness of the films were measured using screw gauge. The UV–Vis spectra were obtained with a UV-2501 PC spectrometer (Shimadzu, UV-2501 PC) in the range of 190–900 nm at 25 °C with a 1 nm sampling interval and 1 nm slit width. Raman spectra were obtained using Raman spectroscopy (Witec alpha300 SR, 488 nm). The thermogravimetric (TGA) analysis of the PUA was measured using TGA analyser (TA Instruments Q50). The measurements were done by heating the films at a rate of 20 °C/min from 100 to 600 °C. The *T*_g_ of the PUA samples were determined by DSC (TA Instruments DSC Q10) at a heating rate of 10 °C/min in the nitrogen atmosphere. The dynamic mechanical behavior of the films was observed by DMA (TA Instrument Q800) with the tensile mode at a heating rate of 50 °C/min in the nitrogen atmosphere. The DMA was measured using a dynamic mechanical analyser (DMA) (TA Instruments, DMA Q800) at a constant frequency of 1 Hz. The sample was heated and cooled from −80 to 120 °C at a rate of 3 °C min^−1^ in a liquid *N*_2_ environment. The measured sample was a rectangular shape with a dimension of 13 mm × 5 mm × 0.3 mm. The mechanical properties of the PUA films were measured using an Instron universal testing machine (Instron model 5943) using a load cell of 500 N at a speed of 100 mm min^−1^ at room temperature. The test specimens were cut into rectangular dimensions of 55 mm × 5 mm (length × width). The gauge length was 5 mm. The four-point probe resistance measurement system (Advance Instrument Technology, CMT-SR2000N) was used to measure the sheet resistance of the stretchable electrode. Keithley 2400 source meter was used to measure the resistance of the stretchable conductor under different strain. A home-built stretching stage was used to stretch the conductor and device for various measurements. The mechanical force was exerted on the device by using a mechanical shaker (Sinocera, Model JZK-20) and the magnitude of the force on the sample was measured by a Force gauge (Sinocera, Model CL-YD-303). An oscilloscope (Trektronix, MDO 3024, impedance = 10 MΩ) was used to measure the voltage output from the SH-TENG, a low noise current pre-amplifier (Stanford Research System, Model SR570, impedance = 4 Ω) was used to measure the current output from the TENG, an electrometer (Keithley 6514 electrometer, input resistance = 200 TΩ) was used to measure the charge transfer.

## Supplementary information

Supplementary Information

Description of Additional Supplementary Files

Supplementary Movie 1

Supplementary Movie 2

Supplementary Movie 3

Supplementary Movie 4

Supplementary Movie 5

## Data Availability

The authors declare that the data supporting the findings of this study are available within the article and its Supplementary Information files. All the other data supporting the findings of this study within the article are available from the corresponding author upon reasonable request.
