# Supplementary material for: Extremely stretchable and self-healing conductor based on thermoplastic elastomer for all-three-dimensional printed triboelectric nanogenerator
Source: Nat Commun. 2019 May 14;10:2158. doi: 10.1038/s41467-019-10061-y (PMC6517406; doi:10.1038/s41467-019-10061-y)
Supplement: Supplementary file 1 — Supplementary Information [file 41467_2019_10061_MOESM1_ESM.pdf]

Supplementary Information

Extremely stretchable and self-healing conductor based on thermoplastic elastomer for all three-dimensional printed triboelectric nanogenerator

Parida et. al.

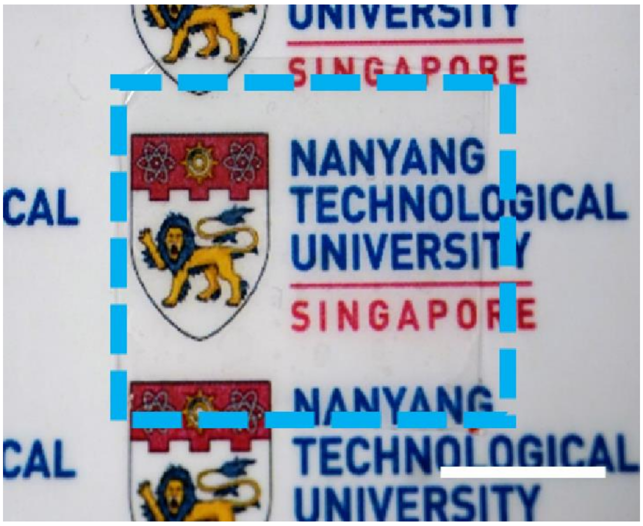

Supplementary Figure 1. Digital photo of the highly transparent polyurethane acrylate (PUA) film. Scale bar: 1 cm.

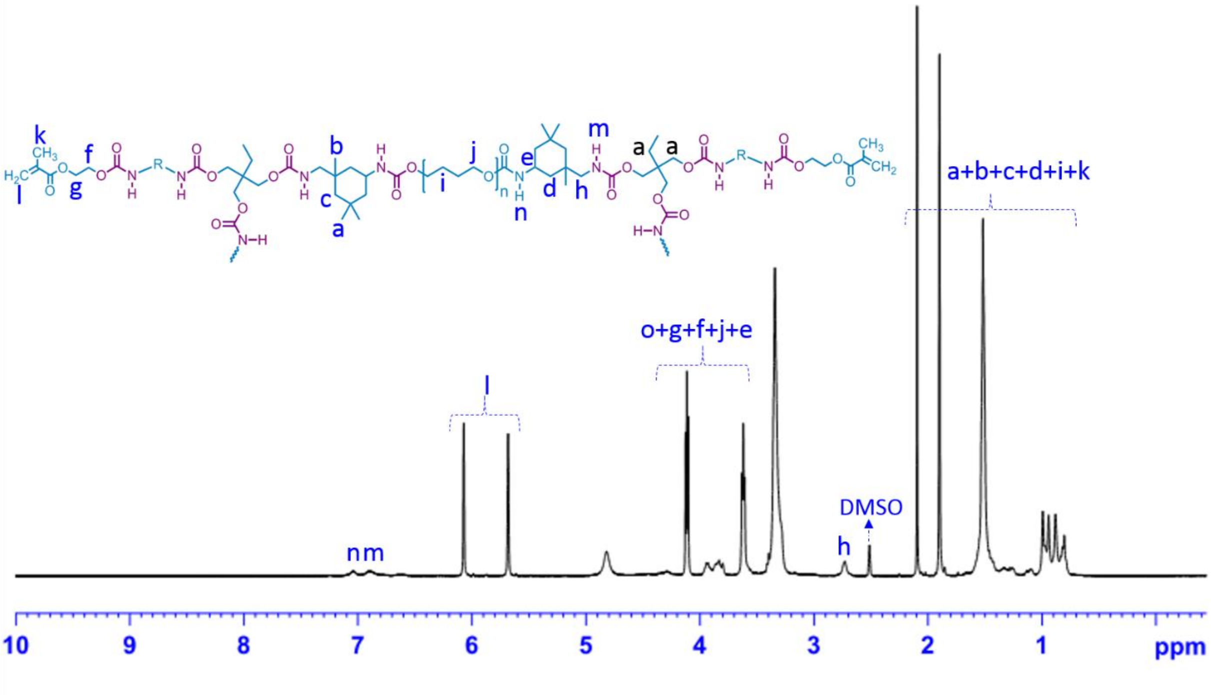

Supplementary Figure 2. ^{1}H NMR spectroscopy showing peaks for existing functionalities in polyurethane prepolymer (PU-NCO) and polyurethane acrylate (PUA) with 20 wt % 2-hydroxyethyl methacrylate (HEMA) (PUA-20%HEMA) at different parts per million. **^{1}H NMR (DMSO):** 0.89 (s, 9H,-CH_3), 0.91(s, 3H, -CH_3), 1.46 (d, 6H, -CH_2), 1.67 (q, 4H,-CH_2-), 2.86(d, 2H, -CH_2-NH-), 3.43 (d, 2H, -CH-O-), 3.98 (s, 4H,-C-CH_2-O-), 8.03 (t, 1H, -N<u>H</u>-C=O).

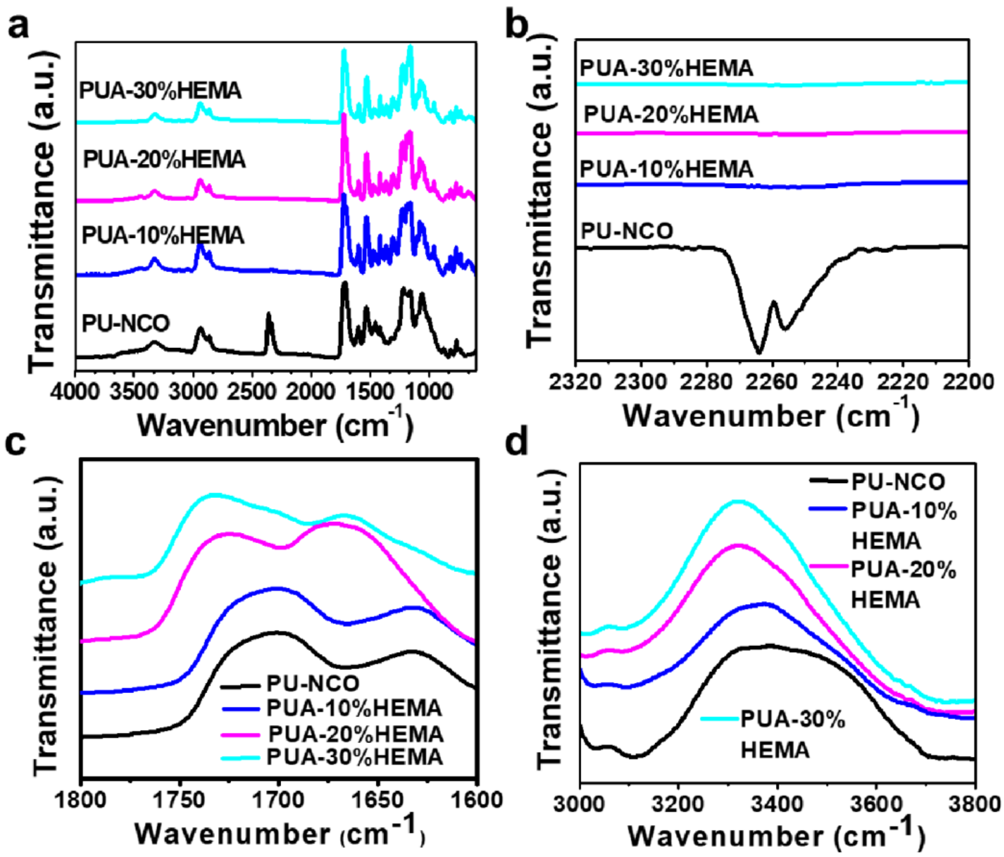

Supplementary Figure 3. FTIR spectra of various polyurethane acrylate (PUA) with different amount of 2-hydroxyethyl methacrylate (HEMA). **a** Complete FTIR spectra of PU-NCO, PUA-10%HEMA, PUA-20%HEMA, and PUA-30%HEMA. **b** Enlarged range for N=C=O stretching band (The NCO peak at 2266 cm^{-1} suggests that the diisocyanate monomer were fully converted to urethane bond). **c** The existence of -C=O stretching (1800-1600 cm^{-1}) zone of different HEMA content. (d) Existence of -N-H (3000-3800 cm^{-1}) stretching zone of different HEMA content.

Supplementary Note 1.

Supplementary Figure 3 shows the FTIR spectra of PU–NCO, PUA–10%HEMA, PUA–20%HEMA and PUA–30%HEMA. Supplementary Figure 3a shows the band between 2800 and 3000 cm^{-1} corresponds to stretching vibrations (symmetric CH_3 stretching: 2873 cm^{-1}; symmetric CH_2 stretching: 2848 cm^{-1}; asymmetric CH_3 stretching: 2951 cm^{-1}; asymmetric CH_2 stretching: 2927 cm^{-1}). The peaks at 1682 cm^{-1} (C–C), 1452 cm^{-1} (CH_2), and 843 cm^{-1} (CH) indicate that the C=C bonds of acrylate propagated in the PU–NCO chains. The absence of the peak at 2266 cm^{-1} from the spectrum of copolymers of PUA–HEMA when compared to the spectrum of the prepolymer (PU–NCO) indicate that the NCO group of the prepolymer has fully reacted with the hydroxyl groups (OH) of HEMA (Supplementary Figure 3b). The peaks at 1720 and 1729 cm^{-1} correspond to the C=O stretching vibrations (hydrogen bonding, Supplementary Figure 3c). The band corresponding to C=O stretching

vibrations is divided into two zones: the free C=O region and the hydrogen-bonded C=O region (Supplementary Figure 3c). The absorption peak at 1728 cm^{-1} is assigned to free urethane (–NH–COO–) carbonyl stretching, whereas the peaks at 1728, 1724, and 1708 cm^{-1} correspond to hydrogen-bonded CO stretching. The absorption peaks at 3333 and 3365 cm^{-1} can be attributed to the NH stretching region (hydrogen bonding, Supplementary Figure 3d).

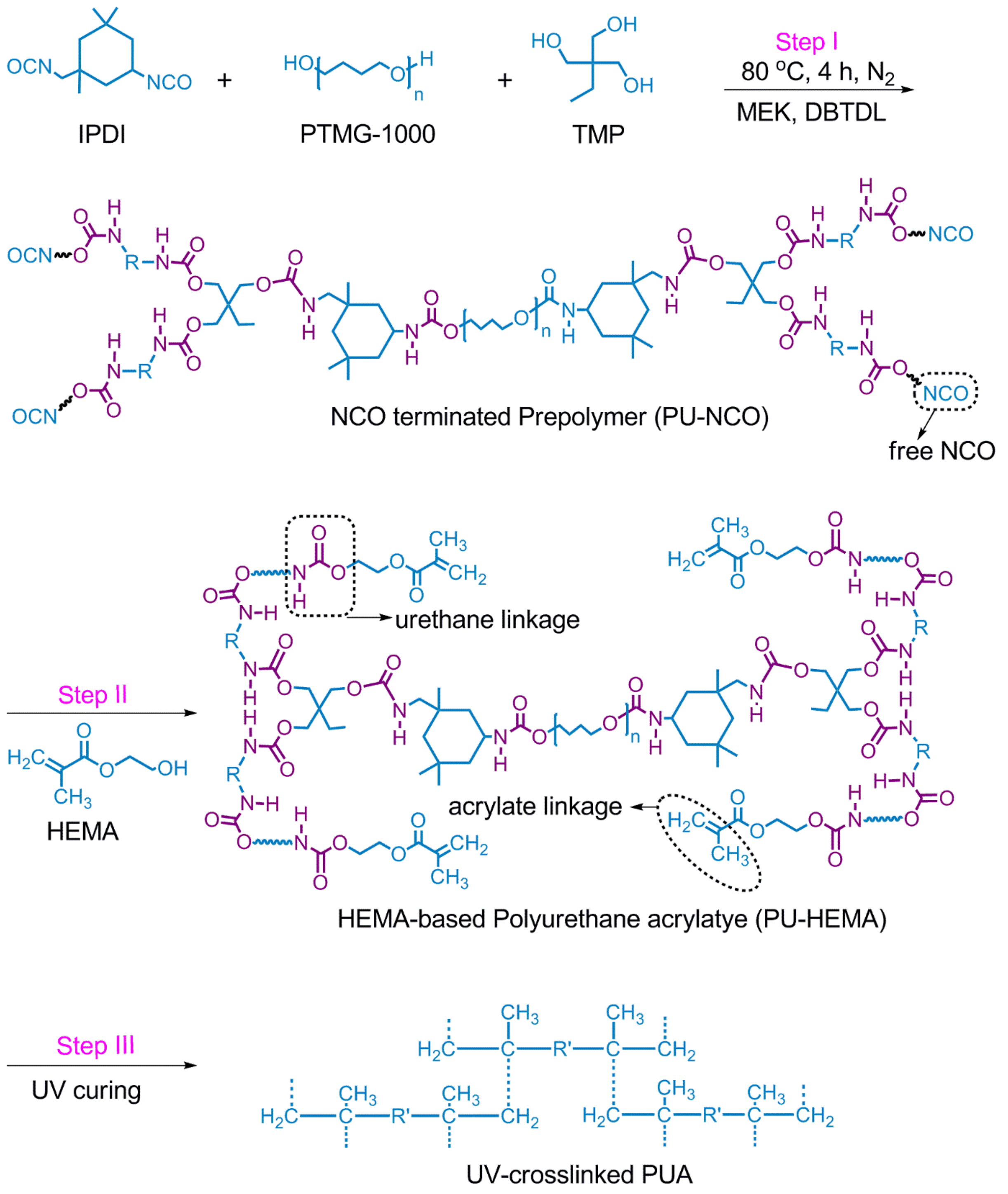

Supplementary Figure 4. Schematic representation of the preparation of polyurethane acrylate via condensation polymerization.

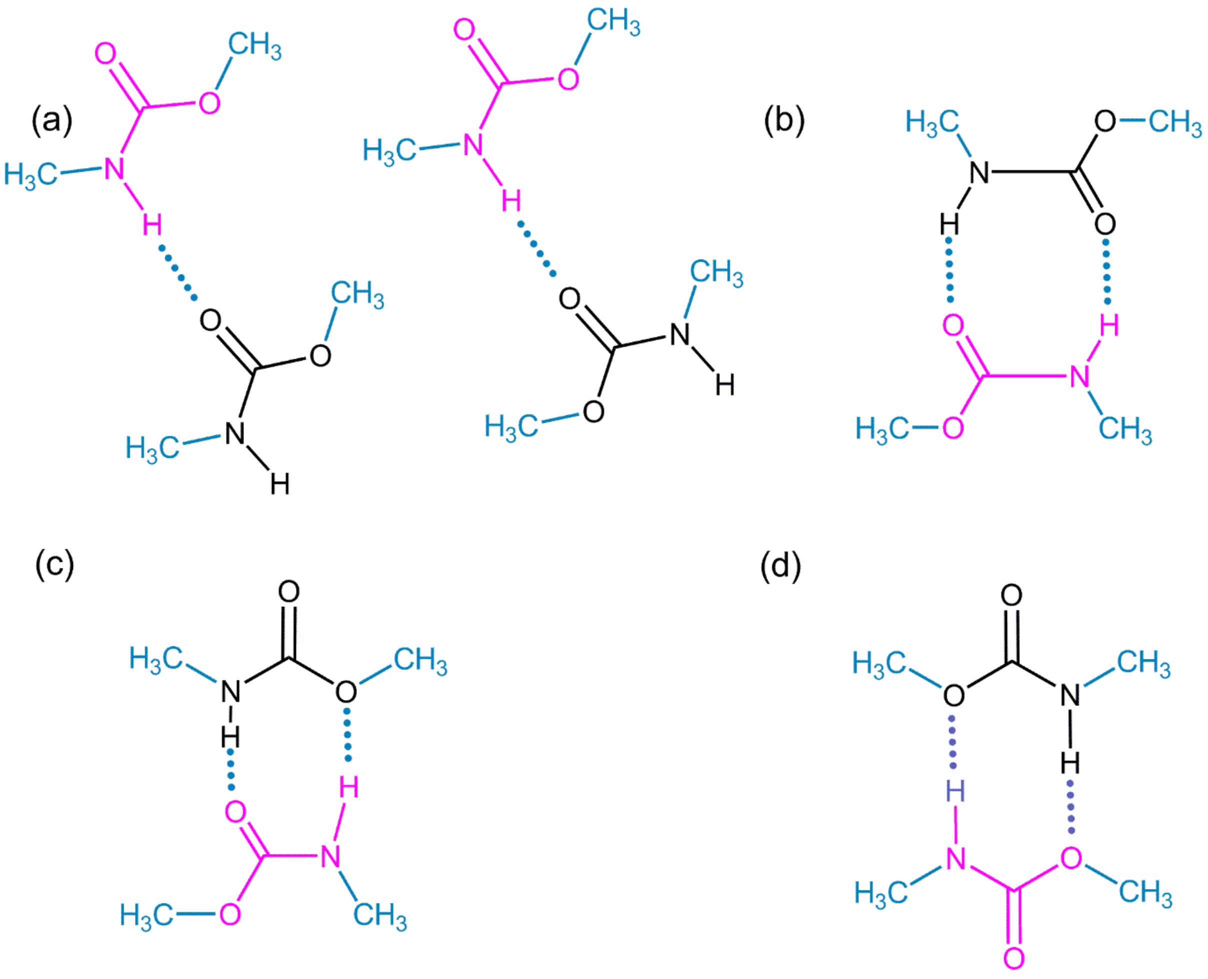

Supplementary Figure 5. Four types of H-bonds in polyurethane acrylate (PUA). a) Two different H-bonded types in each H-bonded complex; b) H-bond dimer formed by two C (t,c) conformations; c)-bond dimer between NH and C=O, alkoxyl; d) H-bonded dimer between NH and alkoxyl oxygen.

Supplementary Note 2.

Generally, polyurethane (PU) consists of hard and soft segments. The hard segments have urethane units [–NH–CO(O)–], which served as the physical crosslinks between soft segments. The interactions between soft segments in PUs are closely related to H-bonds. In our PUA system, the four types of H-bonding interaction are considered to be formed between the NH group and C=O group. As shown in Supplementary Figure 2, all four types deal with the urethane-urethane [–NH–CO(O)–] - [–NH–CO(O)–] interactions. The urethane group provides one H-bond donor and three possible acceptors, and another acceptor of the ether oxygen from soft segments. The presence of the different H bondings in the PUA leads to the microphase separation, that exerts an important effect on the mechanical properties, and molecular aggregation.

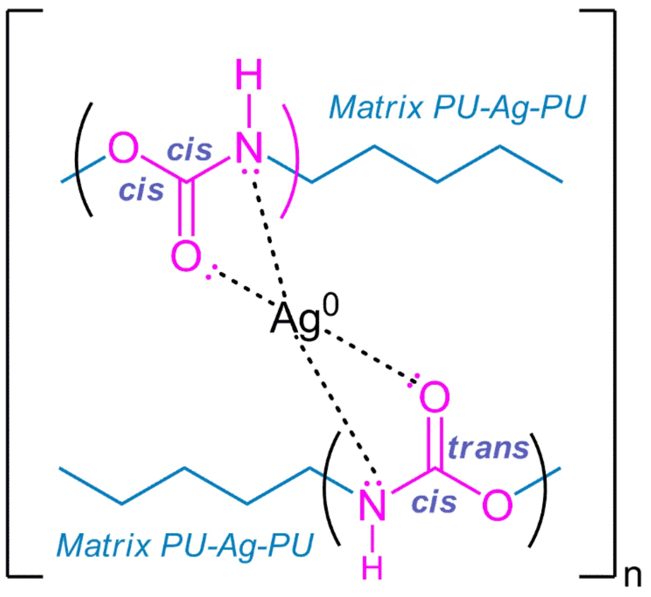

Supplementary Figure 6. Schematic representation of the bonding of polyurethane acrylate (PUA) with the fatty acid on the surface of commercial silver flakes.

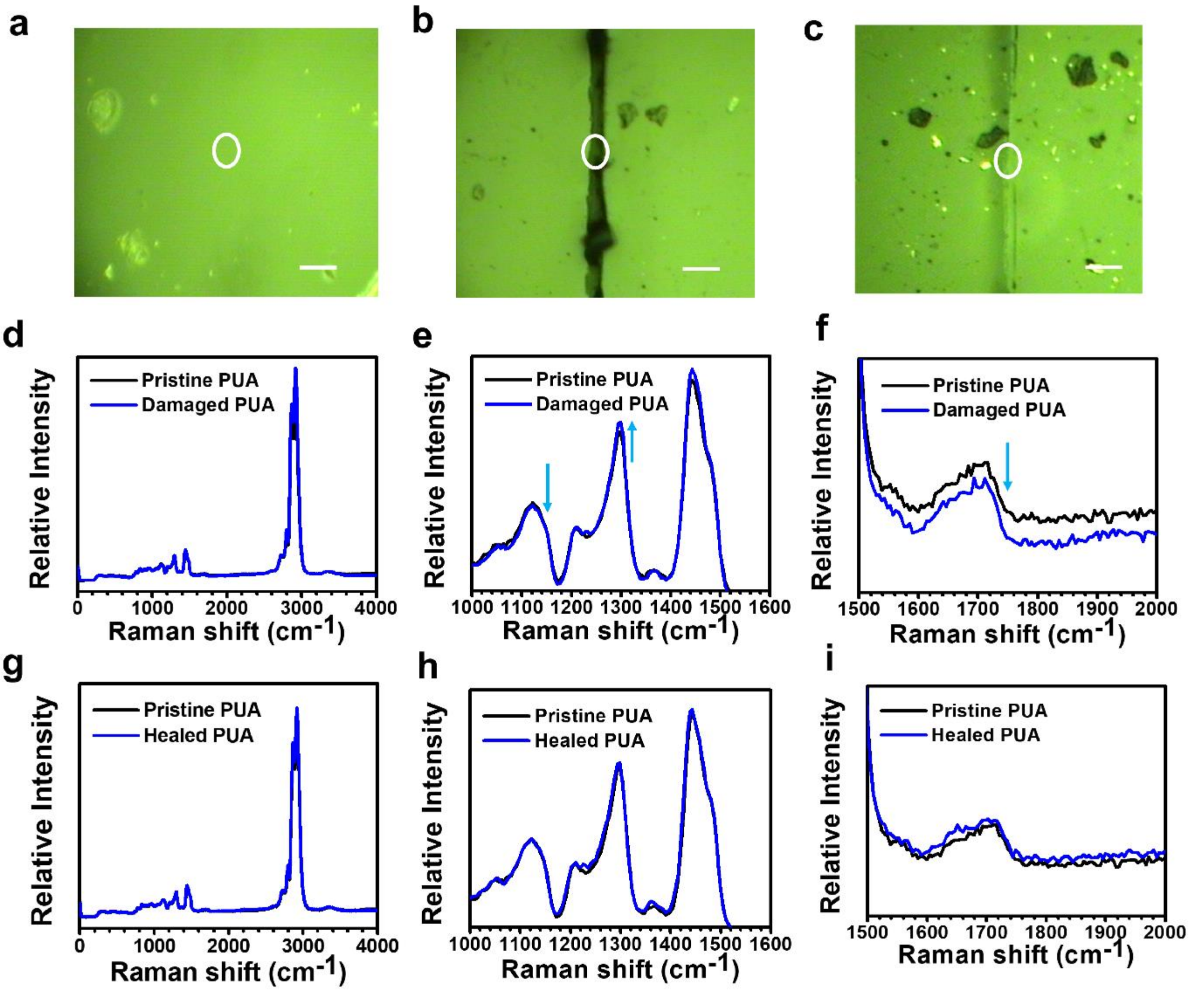

Supplementary Figure 7. Optical image and Raman spectra of polyurethane acrylate (PUA) indicating the self-healing behaviour. Optical image of PUA film **a** Pristine undamaged PUA film, **b** Damaged PUA film, and **c** Optical image of the healed PUA film. Scale bar: 20 µm.The circle indicates the Raman imaging area. **d** Complete Raman spectra of the pristine undamaged PUA film and the damaged PUA film. **e** and **f** Enlarged Raman Spectra of the pristine undamaged PUA film and the damaged PUA film. **g** Complete Raman spectra of the pristine undamaged PUA film and the healed PUA film. **h** and **i** Enlarged Raman spectra of the pristine undamaged PUA film and the healed PUA film.

Supplementary Note 3.

To confirm the role of multiple H-bonds in the healability of the PUA, Raman spectroscopy was carried out to evaluate the molecular structure of PUA networks. For the damaged PUA film, Raman spectra were obtained from the site of damage (by scratching, Supplementary Figure 7b). When the PUA film is damaged, the C-C and C-H band intensities associated with -N at 1290-1305 slightly increases with respect to the C-H and C-C vibrations associated with –O at 1124-1133 cm^{-1} (Supplementary Figure 7d and e). These changes can be attributed to the cleavage of urethane linkages (NH-CO-O). If the cleavage occurs at the urethane (O)C–N bond, formation of –R–NH_2 and –CH_3 or –CH_2– at the cleaved ends of

the polymer chain will occur, and if the cleavages occur at urethane O–C(O) bond, then –R–NH_2 and –R–OH groups will be formed. Mechanical damage also leads to either cleavage or conformational changes of the H-bond (the carbonyl stretching wavenumbers at 1700-1721 cm^{-1} to H-bonded C=O from urethane linkages), thus decreasing the band intensity of the damaged PUA film compared to the pristine non-damaged PUA film (Supplementary Figure 7f). After the PUA film is healed, the spectra are similar to that of the non-damaged PUA film, indicating the recovery of H-bond (the carbonyl stretching wavenumbers at 1700-1721 cm^{-1} to H-bonded C=O from urethane linkages) (Supplementary Figures 7g,h and i). Thus, the breakage and recovery of H-bond suggest the role of H-bonding in healing.

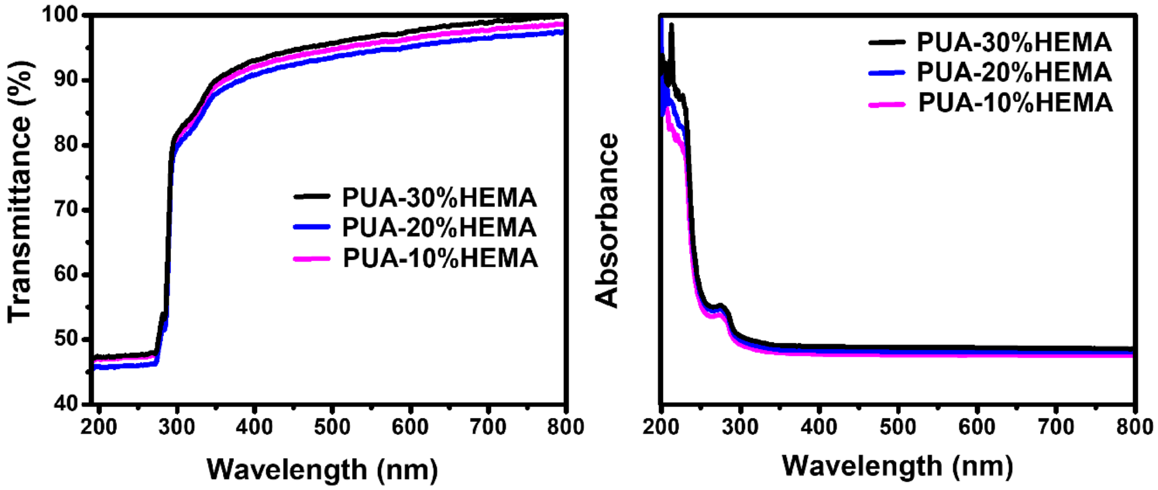

Supplementary Figure 8. Ultraviolet–visible transmittance and absorption spectra of polyurethane acrylate (PUA). **a** Transmittance spectra of PUA with different wt% of 2-hydroxyethyl methacrylate (HEMA). **b** Ultraviolet–visible absorption spectra of samples with different wt% of HEMA.

Supplementary Note 4.

Supplementary Figure 8a shows the UV-Vis transmittance spectra of the PUA with different concentration of HEMA. The PUA film has a transparency of 97% in the region of 500-800 nm. The transmittance value (at 500 nm) for PUA-HEMA 10%, PUA-HEMA 20% and PUA-HEMA 30% samples, are 94.6%, 95.1% and 95.6% respectively. This relative increment of the transmittance is attributed to the strong shielding effect of HEMA in PUA. The absorption intensity of PUA in the range of 200-300 nm, can be attributed to the urethane groups (Supplementary Figure 8b). No significant increase in the absorbance peak with an increase in the HEMA content indicates that the HEMA incorporation does not affect the degree of polymerization after UV-curing.

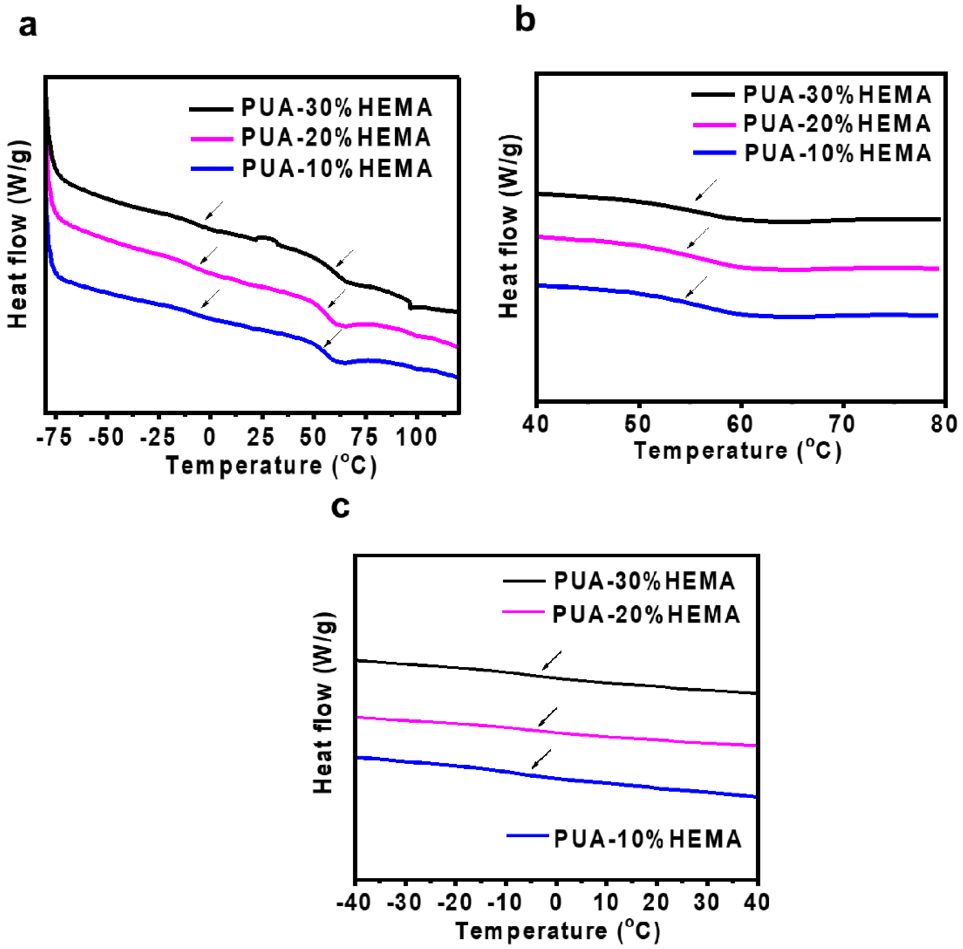

Supplementary Figure 9. Differential scanning calorimetry (DSC) analysis of polyurethane acrylate (PUA) with different amount of 2-hydroxyethyl methacrylate (HEMA). **a** Complete DSC spectrum of the PUA-10%HEMA, PUA-20%HEMA, and PUA-30%HEMA. **b** Enlarged range for first transition. **c** Enlarged range for second transition.

Supplementary Note 5.

Supplementary Figure 9a shows the DSC thermograms of the PUA films with 10, 20 and 30 wt% HEMA. As shown in Supplementary Figure 9a, PUA-10%HEMA and PUA-20%HEMA indicate that the no melting or crystallization shift corresponding to the soft segment has been observed in the DSC curves, indicating the crystalline domains of PTMG are impaired at the time of crosslinking reactions. The melting peaks are absent which can be correlated with viscoelastic nature of PUA. In the Supplementary Figure 9c, the glass transition is clearly visible at 58.62 to 63.11 °C with an increase in the OH number of the HEMA from 10 to 30% (Supplementary Table 1). A weak glass transition at about

-4.45 to -5.12 °C was also observed for all our samples, as illustrated in Supplementary Figure 9b. The identification of this peak is not clear. Such a weak transition has usually been identified due to the movements of a chain section containing the urethane group attached to a crosslinker [1]. The T_g values of crosslinked PUs are affected by several factors, including the relative amounts of the soft and hard segments, the crosslinking density, and the amount of hydrogen bonding [1]. The T_g of both (T_{gss} and T_{ghs}) of the PUA increased upon increasing the content of HEMA, presumably because the high rigidity of the PUA chain reduced the segment mobility and increasing hydrogen bonding.

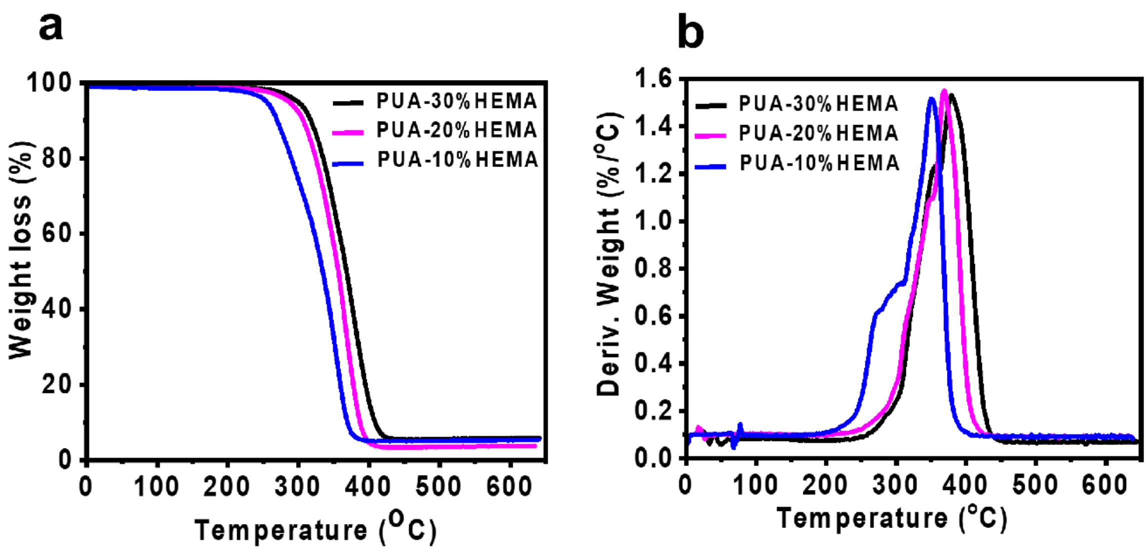

Supplementary Figure 10. Thermogravimetric analysis (TGA) of polyurethane acrylate (PUA) with different amount of 2-hydroxyethyl methacrylate (HEMA). **a** TGA spectrum of the PUA-10%HEMA, PUA-20%HEMA, and PUA-30%HEMA. **b** Derivative of TGA spectrum of PUA-10%HEMA, PUA-20%HEMA, and PUA-30%HEMA.

Supplementary Note 6.

The thermal stability of the PU-NCO and PU-X%HEMA were investigated as a function of temperature from 50 to 650 °C in a nitrogen atmosphere. As shown in Supplementary Figures 10 a and b, all the PUA films showed two stages of decomposition processes. The first stage of decomposition of the PUA films was observed in the temperature range of 262 to 279 °C, and the temperature of maximum thermal degradation (T_{max1}) is 298 to 350 °C (Supplementary Table 1). This is primarily due to the decomposition of the urethane bonds. The 50% weight loss in the degradation processes of the PUA films was observed in the temperature range of 354to 365 °C, and the T_{max2} is 353 to 374 °C. This can be attributed to the

degradation of soft segments from the polyether. When the OH number of the HEMA increases from 10 to 30 wt%, significant thermal stability can be observed, probably because of the competition between the increase in the crosslink density and the higher content of labile hard segments.

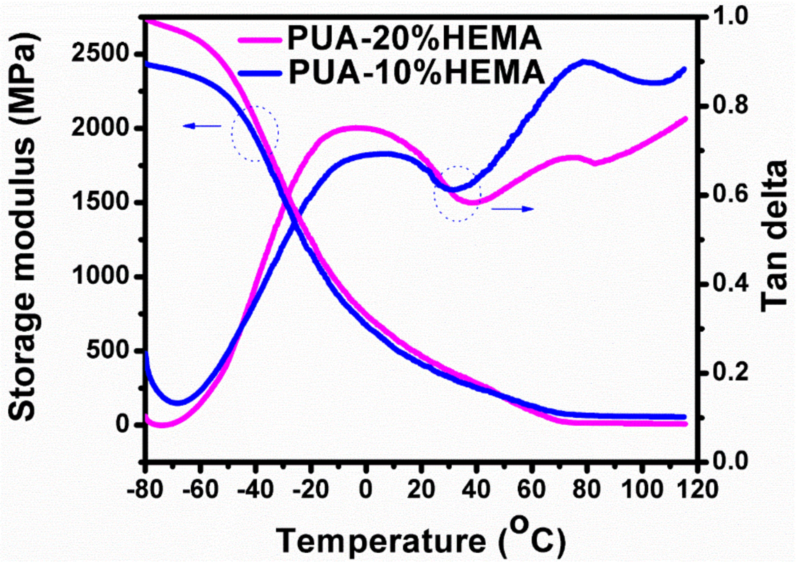

Supplementary Figure 11. Storage modulus and loss tangent delta of polyurethane acrylate (PUA) with different amount of 2-hydroxyethyl methacrylate (HEMA) as a function of temperature measured using dynamic mechanical analysis. (Storage modulus and tan delta concluded that when the temperature is lower than T_g, all the PUA show higher E' values with a small decrease. At a higher temperature it displays a sharp decrease due to molecular mobility which could allow stored energy as a mechanical restoring force that brings the materials to the original state.

Supplementary Note 7.

Dynamic mechanical analysis (DMA) was used to investigate the thermal transition and viscoelastic behaviour of the PUA films. As shown in Supplementary Figure 11, the PUA film shows two phase transitions; the soft segment glass transition temperature and the hard-segment glass transition temperature. The T_g values of these two phases depend on the chemical composition, phase separation and mixing, and the molecular weight of these two phases. When the HEMA content of the PUA was increased from 10 to 20 wt%, there was a shift in the T_{gss} and T_{ghs} from -8.25 to -7.92 °C and 74.33 to 79.72 °C, respectively (Supplementary Table 1). The shifts in T_{gss} and T_{ghs} can be attributed to the increase in intermolecular interactions. With the incorporation of higher content of HEMA, the number of urethane connections increases, thus increasing the intermolecular interactions. As indicated by Supplementary Figure 11, the elastic storage modulus of the PUA increased from 2455 MPa to 2749 MPa with the increase in the HEMA content from 10 to 20 wt%. This increment can be attributed to the increase in the crosslinking density.

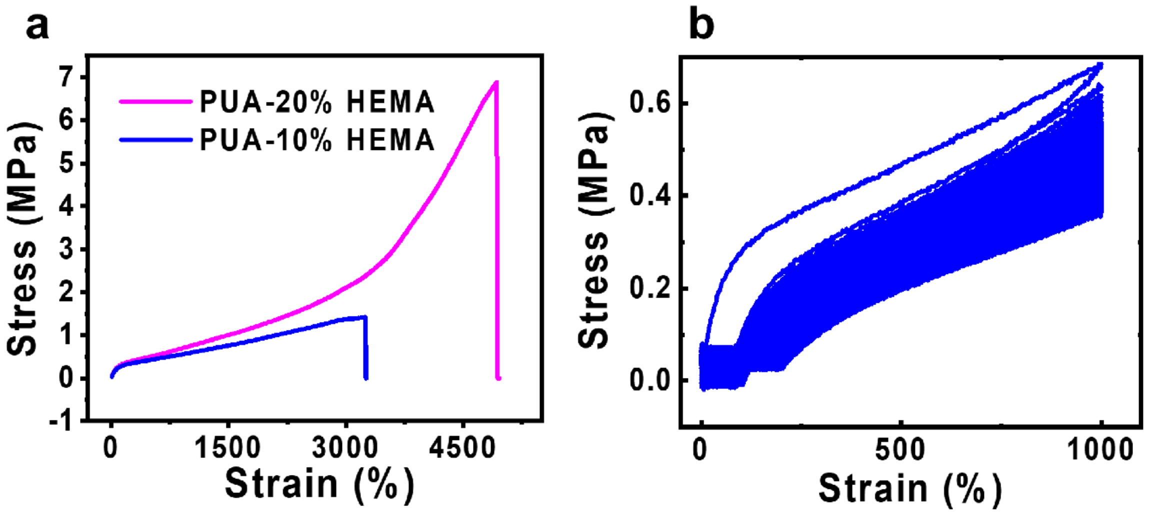

Supplementary Figure 12. Mechanical stress-strain measurement of polyurethane acrylate (PUA) with different amount of 2-hydroxyethyl methacrylate (HEMA). **a** Tensile stress-strain behaviour of the PUA-10%HEMA and PUA-20%HEMA. **b** Cyclic stability test of the PUA- 20%HEMA. The sample is subjected to cyclic tensile strain upto 1000 % for 1000 cycles. Deformation rate for all measurements: 100 mm.min^{-1}.

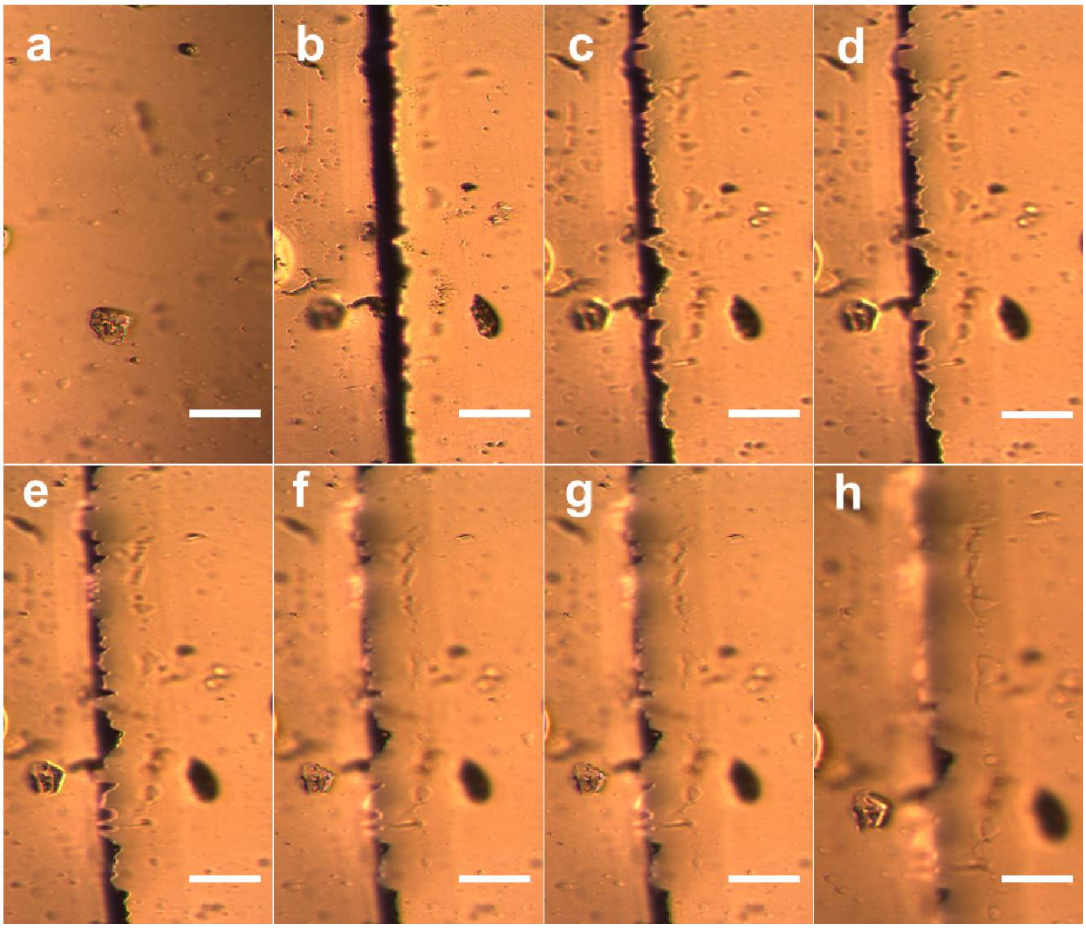

Supplementary Figure 13. Polarized optical micrograph demonstrating the self-healing capability of the two bifurcated polyurethane acrylate (PUA) film. **a** undamaged film, **b** PUA film after complete bifurcation, **c** healing after 4 h at 100 ^{o}C, **d** healing after 8 h at 100 ^{o}C, **e** healing after 12 h at 100 ^{o}C, **f** healing after 16 h at 100 ^{o}C, **g** healing after 20 h at 100 ^{o}C, **h** the visible damage could be totally eliminated after healing for 24 h under 100 ^{o}C. Scale bar: 50 μm.

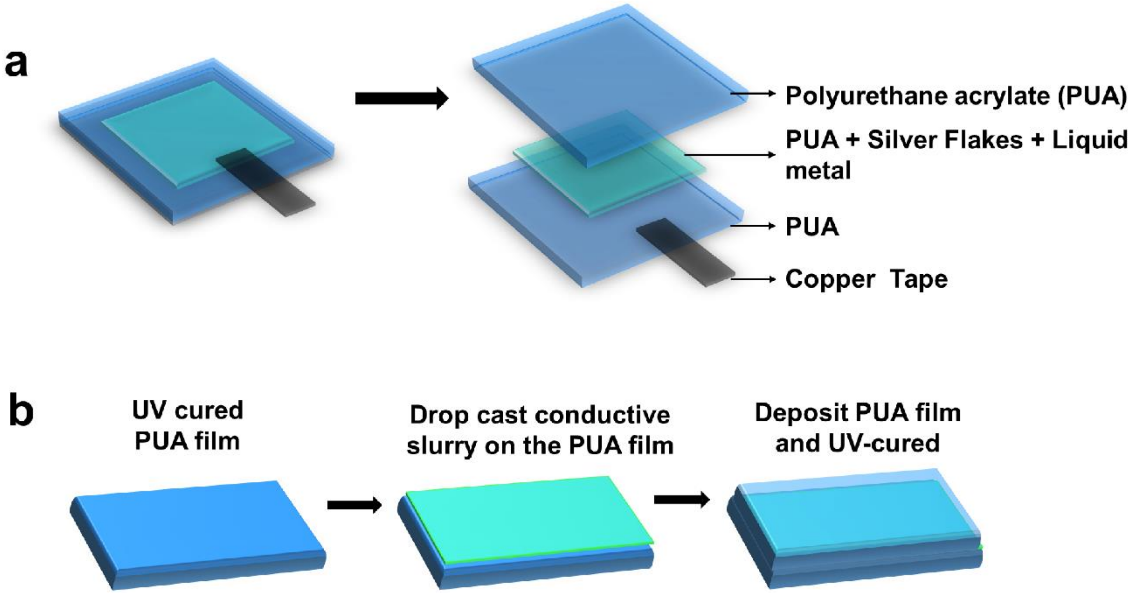

Supplementary Figure 14. Schematic representation of the fabrication of the stretchable and healable triboelectric nanogenerator (SH-TENG). **a** Schematic representation of the SH-TENG, showing the different layers. **b** Schematic representation of the fabrication process of the SH-TENG. The conductive slurry (Polyurethane acrylate (PUA) + silver flakes + liquid metal) was drop-casted on the ultraviolet-cured (UV-cured) PUA films. Subsequently, one more layer of PUA was deposited and UV-cured to obtain the sandwiched structure.

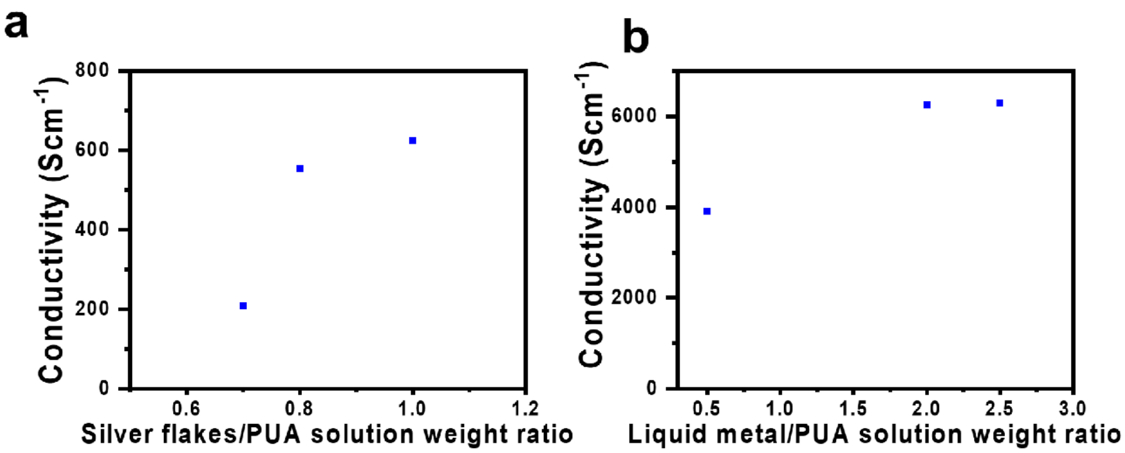

Supplementary Figure 15. Conductivity of the stretchable conductor to optimize the weight ratio of polyurethane acrylate (PUA), silver flakes, and liquid metal. **a** Conductivity of the conductor by varying the weight ratio of silver flakes and PUA, without the addition of liquid metal. **b** Conductivity of the conductor by varying the weight ratio of liquid metal and PUA, keeping the weight ratio of silver flakes and PUA fixed at 1:1. The amount of PUA, silver flakes and liquid metal particles was optimized in the weight ratio of 1:1:2.

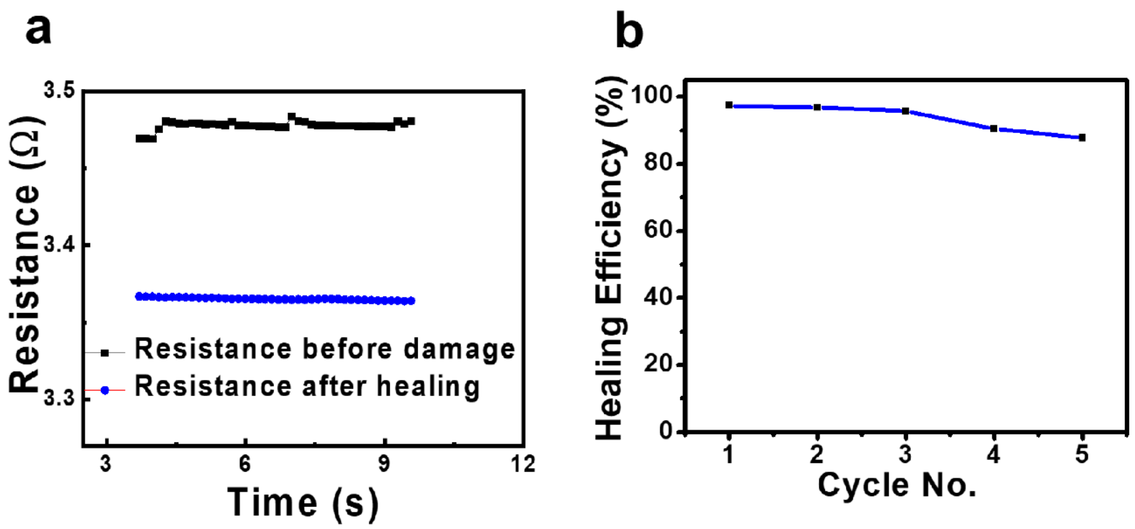

Supplementary Figure 16. Electrical measurements to demonstrate the healing of the conductor. **a** Change in the resistance of the stretchable conductor demonstrating the healing process. **b** Change in the healing efficiency of the stretchable conductor for 5 cycles of healing process.

Supplementary Note 8.

To demonstrate the change in the resistance of the conductor during the self-healing process, the conductor was bifurcated into two parts; then manually attached and heated for 24 h at 100 °C and subsequently allowed to heal for 24 h at room temperature (30 °C). The electrical self-healing efficiency of the healable conductor is calculated by measuring the resistance of the conductor before and after healing, using Supplementary equation 1. Supplementary Figure 16a represents the resistance of the conductor before and after the healing process.

$$\eta = \frac{R_s}{R_i} \times 100 \qquad (1)$$

Where, R_i is the initial resistance of the stretchable conductor before mechanical damage and R_s is the resistance of the stretchable conductor after healing. The calculated electrical self-healing efficiency of the conductor is 96.0 %. The recovery of the electrical resistance of the stretchable conductor after 5 cycles of healing process (Supplementary Figure 16b), indicates the self-healing behaviour of the stretchable conductor.

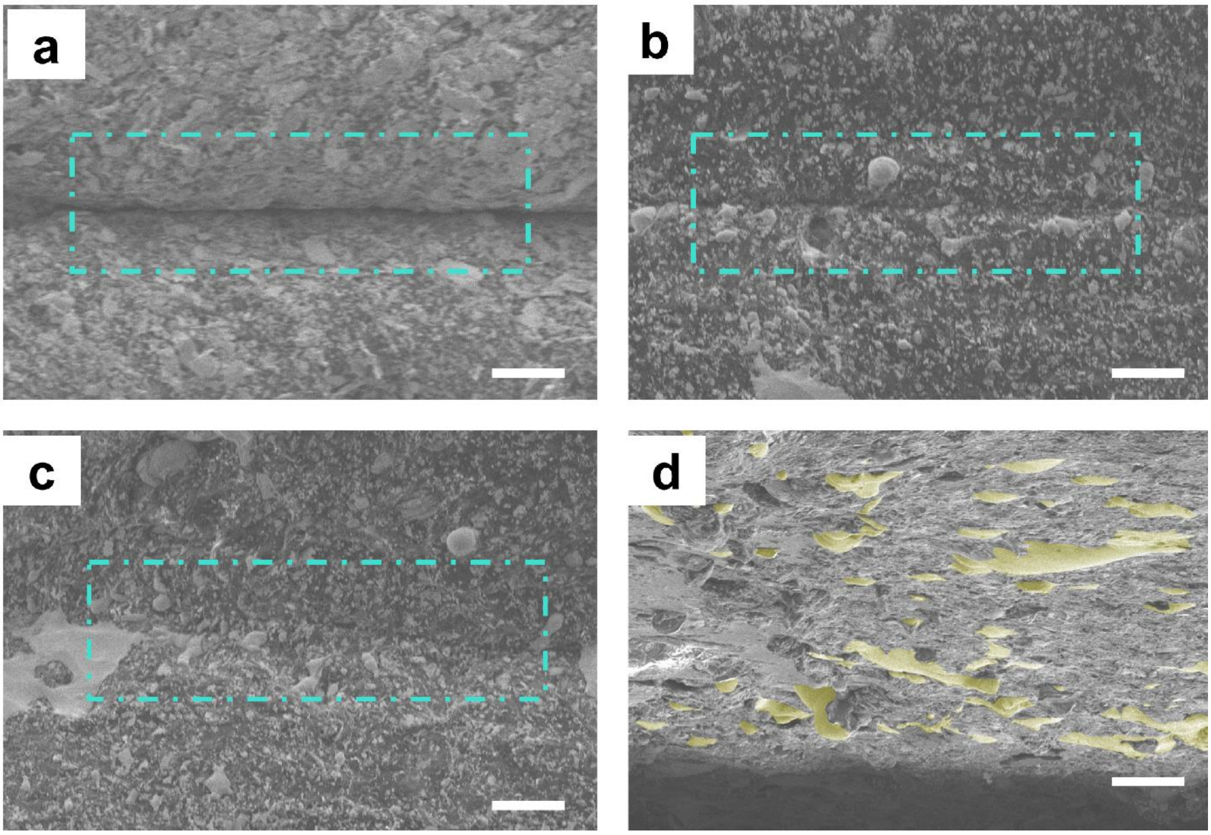

Supplementary Figure 17. FESEM image showing the microstructure changes of the stretchable conductor (polyurethane acrylate+silver flakes+liquid metal) during different time of healing. FESEM image of **a** damaged conductor, **b** conductor healed for 12 hrs at 100 ^{o}C, **d** conductor healed for 24 hrs at 100 ^{o}C. Scale bar: 30 µm. **d** Cross-section FESEM image of the damaged/cut part of the conductor. Scale bar : 10 µm. The yellow portion marked in the FESEM image indicates the liquid metal.

Supplementary Note 9.

The liquid metal would not leak out when the device is damaged/cut as the liquid metal particles are embedded in the polymer matrix. The liquid metal EGaInPs particles always have a thin layer of Ga_2O_3[45]. As an optimized small amount of liquid metal used, they are well dispersed in the PUA matrix. PUA matrix is infused with silver flakes, which encompasses the liquid metals, thus preventing the aggregation. After the conductor is damaged, the liquid metals can be seen well distributed in the matrix without being 'leak out' (Supplementary Figure 17d). If a higher amount of liquid metal is used, aggregation will occur due to high surface tension thus the liquid metal will drop out of the device when damaged or cut.

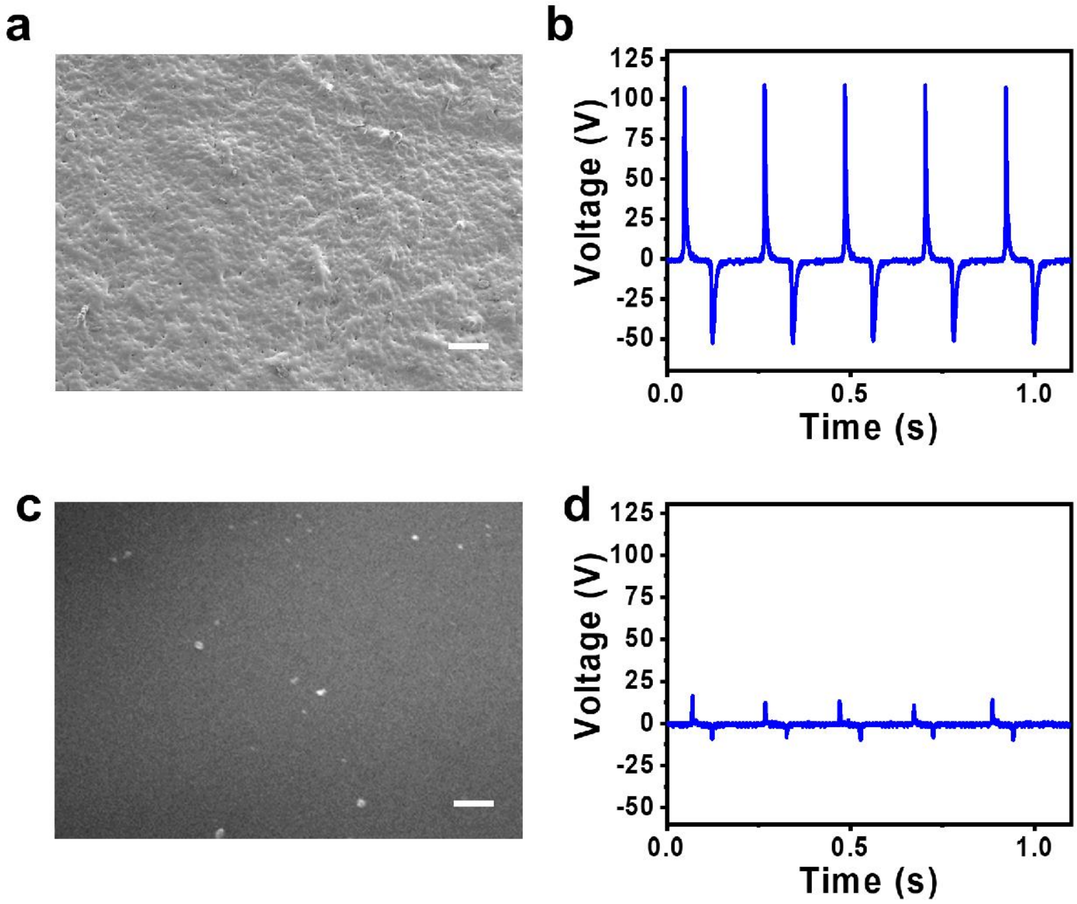

Supplementary Figure 18. FESEM image and energy harvesting performance of the stretchable and healable triboelectric nanogenerator (SH-TENG) with and without microstructures on the surface of polyurethane acrylate (PUA). **a** FESEM image of the microstructures on the surface of PUA. Scale bar: 100 μm. **b** The voltage output of the SH-TENG (force: 40 N, frequency: 5 Hz) with microstructures on the surface of PUA. **c** FESEM image of the PUA without microstructures on its surface. Scale bar: 10 μm. **d** The voltage output of the SH-TENG (force: 40 N, frequency: 5 Hz) without the microstructures on the surface of PUA.

Supplementary Note 10.

To increase the surface area of the PUA, microstructures were formed on the surface of PUA by drop casting the PUA solution onto a sandpaper (Grit = P2500, used as a template). As can be seen, from the Supplementary Figure 18, the voltage output of the PUA with the microstructure is higher than the PUA without the microstructure. The enhancement of the voltage output can be attributed to the increased surface area, which increases the overall surface charge thus improving the voltage output.

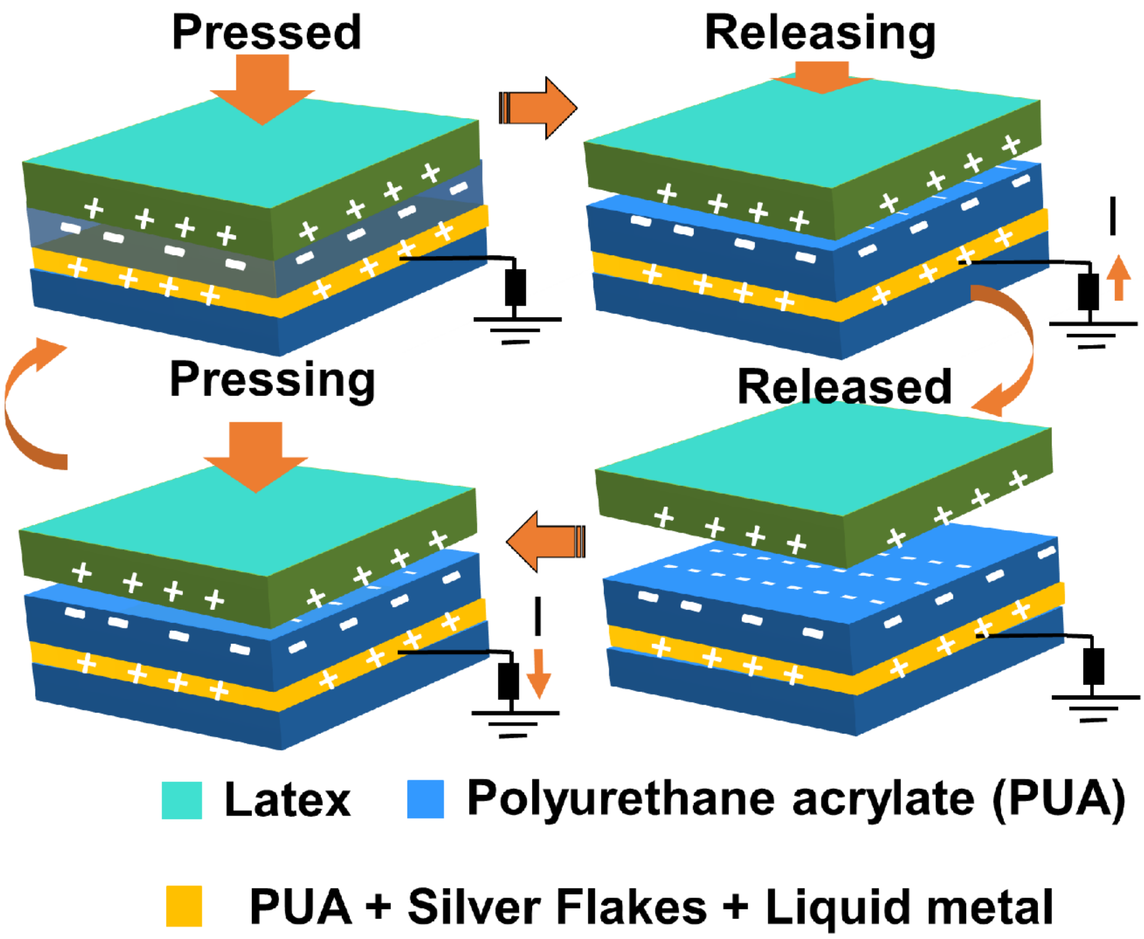

Supplementary Figure 19. Schematic diagram to illustrate the working mechanism of the stretchable and healable triboelectric nanogenerator (SH-TENG).

Supplementary Note 11.

Equation for potential difference and the short circuit charge

The voltage output of the triboelectric nanogenerator is described by Supplementary equation 2 and the short-circuit charge is described by Supplementary equation 3. Where, (V_{oc}) is the open-circuit voltage, (Q_{sc}) is the short-circuit charge quantity, σ is the electrostatic charges density generated on the VHB surface, A is the contacting area between the two polymeric films, C_o is the capacitance of the device.

$$V_{oc} = -\frac{\sigma A}{2C_o} \quad (2)$$

$$Q_{sc} = -\frac{\sigma A}{2} \quad (3)$$

Supplementary Note 12.

Mechanism behind the change in the output performance of SH-TENG upon changing the applied deformation

As the mechanical pressure exerted on the SH-TENG is increased, it will lead to more deformations of the microstructures present on the surface of the triboelectric layer (PUA layer) (Supplementary Figure 20b). This will increase the surface area, thus improving the surface charge density and hence the output voltage. A further increase in the applied pressure creates larger deformations of the interfacial surface causing a further increase in the surface charge density and the output voltage (Supplementary Figure 20c). At a certain pressure when the two materials are in complete contact with each other (where no further macroscopic deformation is possible), the output voltage saturates. Thus, the total attainable interfacial surface area depends on the exerted pressure and the structures present on the surface of the polymer.

The voltage output of the TENG depends on the surface charge density, which in turn depends on the surface area. Due to the formation of microstructures on the surface of the triboelectric layer, the surface area significantly increases, which further increases the surface charge density. The high output voltage can be attributed to the high surface charge density of the triboelectric material. In the TENG device, two layers are separated by a space; thus the internal impedance of the device is very high. Due to the high internal impedance, the voltage output is high, and the current output is low. There have been several reports with high voltage output as can be seen from Supplementary table 4.

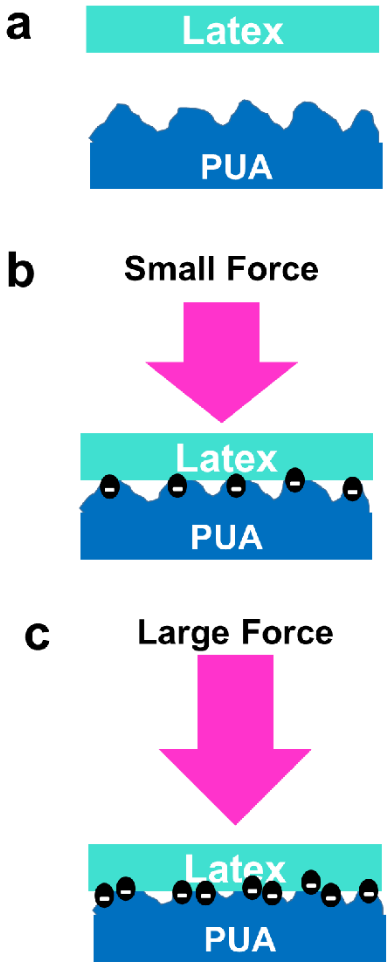

Supplementary Figure 20. Schematic diagram to illustrate the mechanism of the the stretchable and healable triboelectric nanogenerator (SH-TENG) explaining the change in the output performance of SH-TENG upon changing the applied mechanical force. Polyurethane acrylate (PUA) and Latex is used as the triboelectric material. **a** Schematic diagram illustrates the schematic of the device before the mechanical force is applied. **b** Schematic diagram illustrates the schematic of the device when a smaller force is applied. **c** Schematic diagram illustrates the schematic of the device when a larger force is applied.

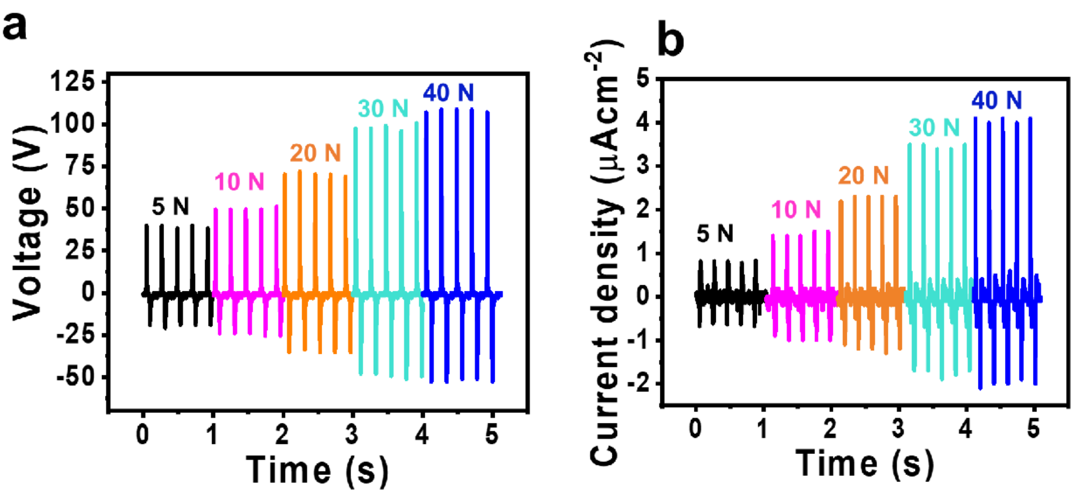

Supplementary Figure 21. Evaluation of the energy harvesting performance of the stretchable and healable triboelectric nanogenerator (SH-TENG) when subjected to different magnitude of mechanical impact at a constant frequency of 5 Hz. **a** Voltage output (V_{op}). **b** Current density (I_s).

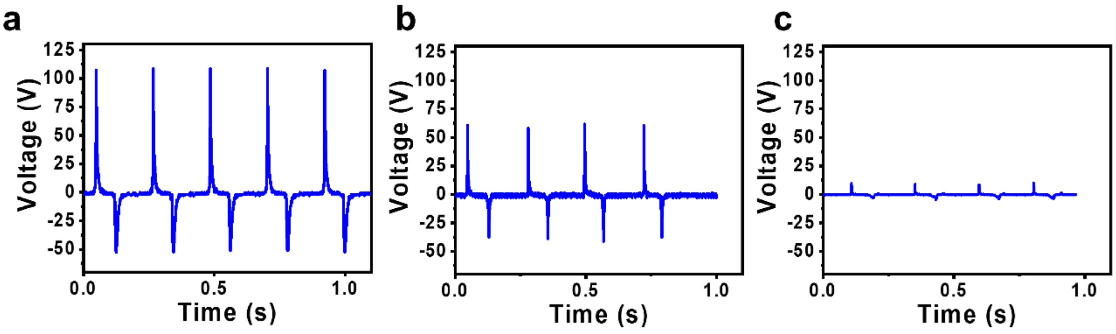

Supplementary Figure 22. Variation of the voltage output of the stretchable and healable triboelectric nanogenerator (SH-TENG) with different thickness of the triboelectric layer (polyurethane acrylate (PUA) layer). **a** The thickness of PUA layer = 200 μm. **b** The thickness of PUA layer = 1 mm. **c** The thickness of PUA layer = 5 mm.

Supplementary Note 13.

As can be seen from Supplementary Figure 22 the voltage output decreases with the increase in PUA thickness of the SH-TENG. The increase in the thickness will decrease the capacitance of the device, thus decreasing the surface charge density. As indicated by Supplementary equation (2) the voltage output of the triboelectric nanogenerator depends on the surface charge density; thus the voltage output decreases with the increase in the thickness of the device.

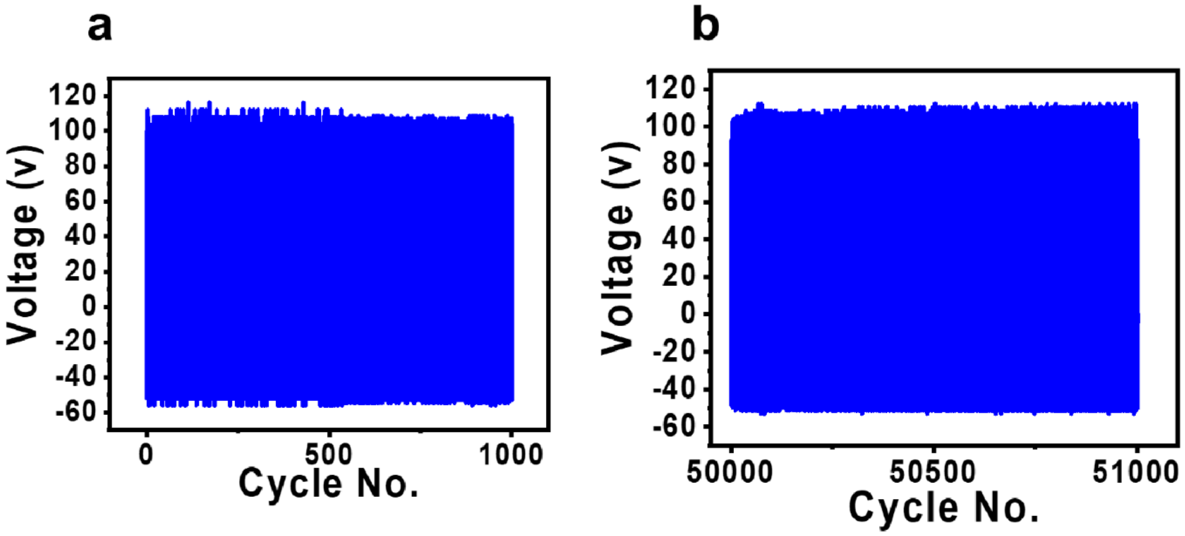

Supplementary Figure 23. Performance stability of the stretchable and healable triboelectric nanogenerator (SH-TENG) for 50000 cycles of mechanical impact at a frequency of 5 Hz. **a** Voltage output of the SH-TENG for the first 1000 cycles of mechanical impact. **b** Voltage output of the SH-TENG for cycle no. 50000 to 51000.

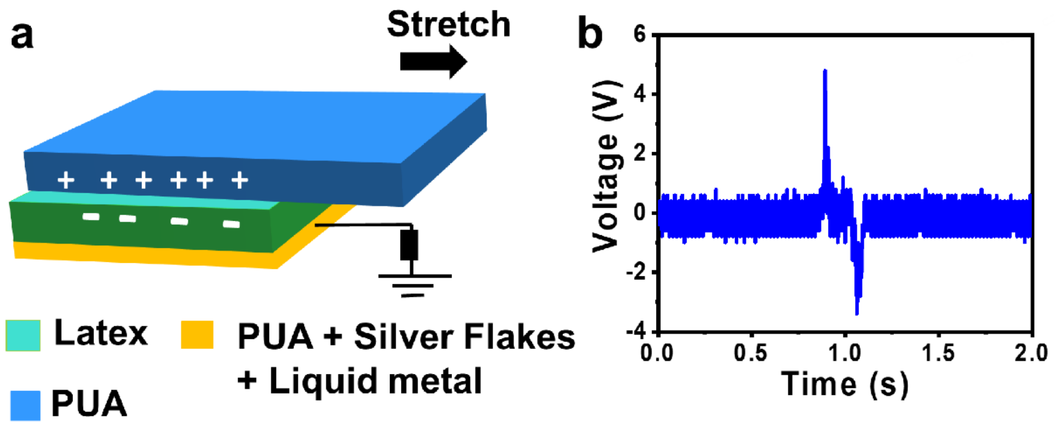

Supplementary Figure 24. Schematic diagram and energy harvesting performance of the lateral sliding mode triboelectric nanogenerator. **a** Schematic diagram of the lateral sliding mode triboelectric nanogenerator. **b** The voltage output of the lateral sliding mode triboelectric nanogenerator (when only the polyurethane acrylate (PUA) layer slides over the Latex layer by stretching PUA to 200 % of its length at a rate of 100 mm/min).

Supplementary Note 14.

In addition to the single electrode mode, lateral sliding mode TENG is demonstrated. As shown in Supplementary Figure 24a, the PUA layer is fixed on the left end and a lateral stretch sliding action is used to exert the frictional triboelectric effect. The pulling tensile stress is equivalent to 0.3 MPa (determined from the stress-strain curve (Fig. 1d).

However, the voltage of the device in the sliding mode is low compared to the single electrode mode. In single electrode mode, compressive force is exerted which creates macroscopic deformations at the interface, thus resulting in high surface charge density, and thus high voltage output. However, in the later sliding mode (achieved by stretching the PUA layer), the contact and separation of the two triboelectric layers are not effective, thus generating low surface charge, resulting in low voltage output. Thus, high output performance can be generated from the single electrode mode compared to the lateral sliding mode TENG.

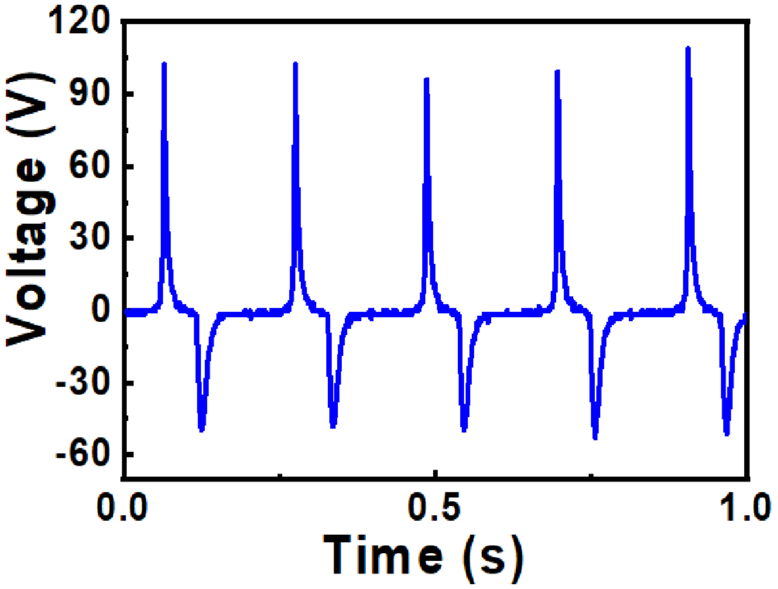

Supplementary Figure 25. The voltage output of the stretchable and healable triboelectric nanogenerator (SH-TENG) (force: 40 N, frequency: 5 Hz) at 0 % axial strain after it is subjected to an axial strain of 2500 %. This shows that the device recovers its energy harvesting performance after strenuous mechanical deformations.

Supplementary Note 15.

In general, the output performance of the TENGs under deformation can be attributed to the following factors, (i) change in the resistance of the electrode, (ii) change in the thickness of the triboelectric layer, (iii) deformations of the microstructures present on the surface of the triboelectric layer, (iv) area of contact between the two triboelectric layers.

If we consider the factors individually,

(i) If the resistance of the electrode increases then the charges generated at the electrode will not be effectively extracted from the triboelectric device. The charges will get dissipated due to the high impedance of the electrode. To validate this, we have measured the voltage output of the TENG by varying the resistance of the electrode.

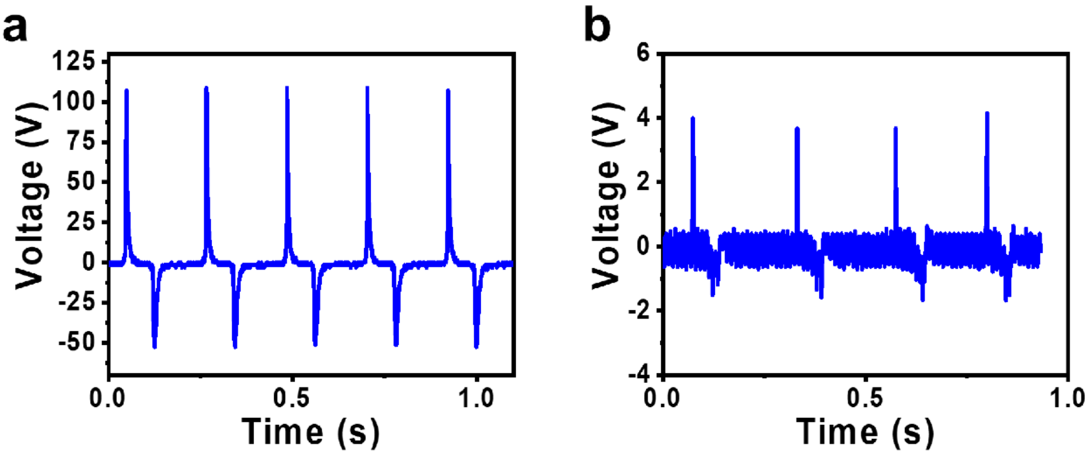

Supplementary Figure 26. The voltage output of the stretchable and healable triboelectric nanogenerator (SH-TENG) with a variation of the resistance of the electrode. **a** Resistance of the electrode = 3 Ω. **b** Resistance of the electrode = 100 kΩ.

As can be seen from Supplementary Figure 26, when the resistance of the electrode increases the output voltage decreases correspondingly. The resistance of the electrode is increased by varying the amount of silver flakes in the PUA electrode in this experiment. Analogously, when the strain is increased the resistance of the conductor increases, thus it will degrade the performance.

(ii) When the triboelectric layer is stretched, the thickness of the layer decreases due to Poisson's effect. The decrease in the thickness increases the capacitance, which will increase the surface charge density of the triboelectric layer, resulting in improved triboelectric performance.
(iii) When the triboelectric layer is stretched, the microstructures on the surface of the triboelectric layers will deform, which will decrease the effective surface area of the triboelectric layer, this will decrease the net surface charge, resulting in lower triboelectric performance.
(iv) As the area of contact between the two triboelectric layer increases the total surface charge increases, thus the performance will improve.

Thus, the net output performance of the TENG when subjected to lateral deformation depends on the most dominating factor, which will vary according to the device design, configuration and electrode.

For the first case in our manuscript, Fig. 4b, when the area of the impacting force (area of the top latex layer, 3 × 3 cm^2) was kept constant, and the measurements were carried out by straining the PUA layer of the SH-TENG device, the output performance (the output voltage and current density (upon application of a mechanical force of 40 N at a frequency of 5 Hz)) decreased with an increase in the

uniaxial strain. When the PUA is stretched the microstructures on the PUA surface deforms, thus decreasing the effective contact area of PUA exposed to the exerted mechanical force. This reduces the net surface charge and the energy-harvesting performance. In the second case (Fig. 4c), when the area of the impacting force (area of the top latex layer) is increased so that it was similar to the uniaxial strain of the device, the output voltage increases. This increase can be attributed to the increment in the surface area of contact between the two triboelectric layer which dominates the overall performance.

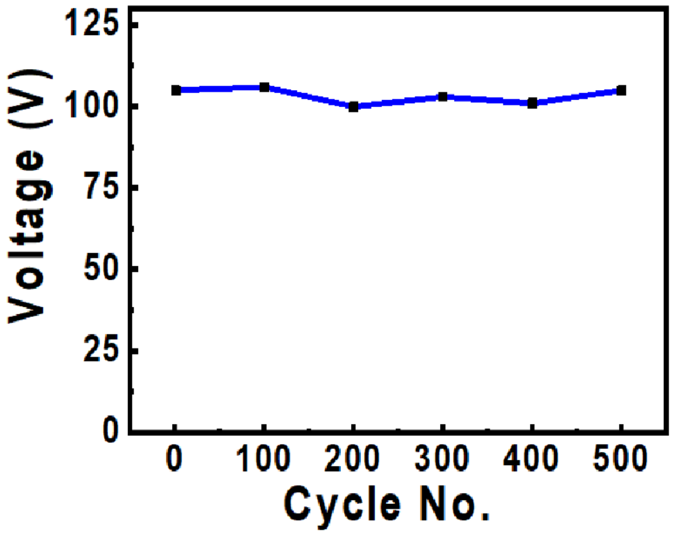

Supplementary Figure 27. Performance of the stretchable and healable triboelectric nanogenerator (SH-TENG) evaluated after every 100 cycles of uniaxial stretching (up to 1000% stretching) for a total of 500 cycles. Deformation rate: 100 mm/min.

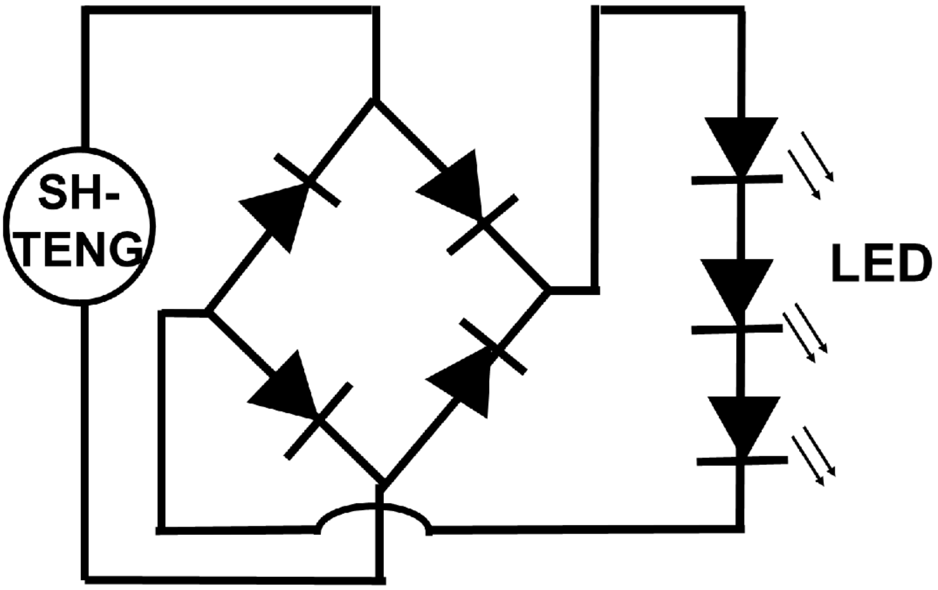

Supplementary Figure 28. Schematic representation of the circuit diagram of powering light-emitting diodes (LEDs) using the stretchable and healable triboelectric nanogenerator (SH-TENG).

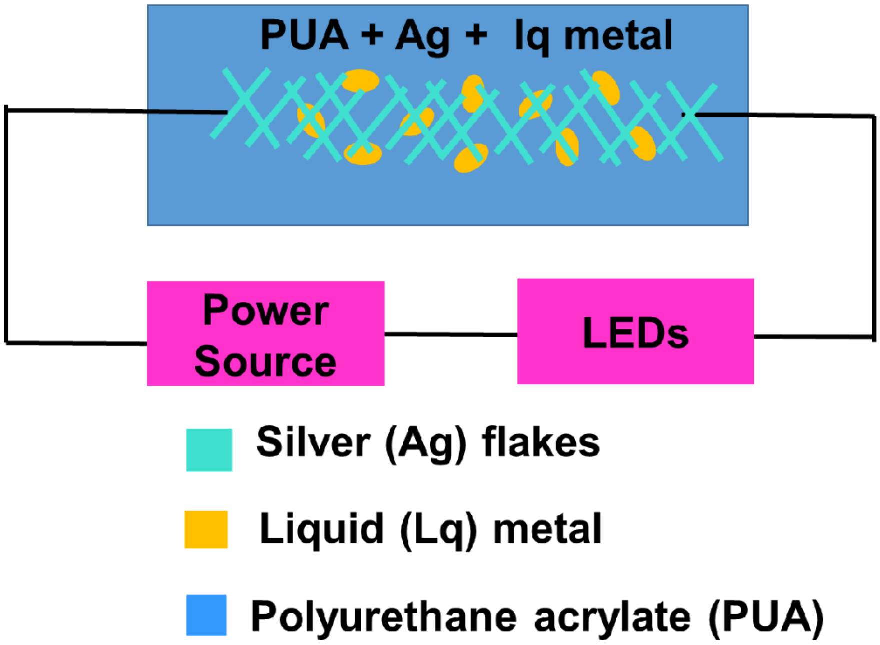

Supplementary Figure 29. Schematic representation of the stretchable conductor of the stretchable and healable triboelectric nanogenerator (SH-TENG) connected in series with a power source and Light-emitting diode (LEDs), to demonstrate that it can sustain high conductivity at extreme stretchability (2500 %).

Supplementary Tables

Supplementary Table 1. Summary of the glass transition temperature of polyurethane acrylate (PUA) with different amount of 2-hydroxyethyl methacrylate (HEMA) from differential scanning calorimetry (DSC), dynamic mechanical analysis (DMA) and thermogravimetric analyser (TGA) studies.

**Polymer**	**[a] DSC (ºC)**		**[b] DMA (ºC)**		**TGA data (ºC)**		
	T_{gss}	T_{ghs}	T_{gss}	T_{ghs}	[c] T_{10}	[d] T_{50}	[e] T_{max}
PUA-10%HEMA	-4.45	58.62	-8.25	74.33	262.72	354.43	353.14
PUA-20%HEMA	-4.78	59.41	-7.92	79.72	272.91	363.08	372.17
PUA-30%HEMA	-5.12	63.11	-	-	279.58	365.77	374.81

[a] Glass transition temperature obtained from DSC; [b] Glass transition temperature obtained from DMA; c) 10% weight loss temperature; d) 50% weight loss temperature; e) Temperature of maximum thermal degradation; T_{gss} - the soft segment glass transition temperature; T_{ghs} - the hard-segment glass transition temperature

Supplementary Table 2. Summary of the mechanical properties of the polyurethane acrylate (PUA) with different healing time.

**Properties**	**Pristine PUA (without mechanical damage)**	**PUA Healed at 100 ºC for 24 h**	**PUA Healed at 100 ºC for 5 h**
Young's Modulus	0.201 MPa	0.201 MPa	0.201 MPa
Elastic limit	4960 %	3700 %	1256 %
Fracture energy	11107 MPa	5011 MPa	557 MPa
Healing efficiency	-	45.11 %	5 %

Supplementary Table 3. Summary of the mechanical properties of various acrylate based self-healing polymer.

Polymer matrix	Segments		TS	EB	Appearance	Reference
	**HSs**	**SSs**	**(MPa)**	**(%)**		
PS/PA-amide	PS	PA-amide	0.26	1570	Non-transparent	[2]
PHEMA	HEMA		0.04	901.8	Non-transparent	[3]
SHWPU	IPDI	PTMG-1000	61.63	310.01	Non-transparent	[4]
TPU	IPDI	PTMG-1000	6.76	923	Yellow transparent	[5]
PUPCL	HDI	PCL	10	35	Non-transparent	[6]
PUU	PPGTD	PUU-g-C_3N_4 NSs	2.04	1503	Non-transparent	[7]
WPUs	LDI	PEG-1450	29.3	954	Non-transparent	[8]
PU	2,4-TDI	PTMG-1000	1.31	380	Non-transparent	[9]
PU-PEGMA	IPDI	PEGMA	3.8	2000	Transparent	[10]
PU	HDI	ε-caprolactone	16.8	137.5	Semitransparent	[11]
**PU-HEMA**	**IPDI**	**PTMG-1000**	**7**	**5000**	**Transparent**	**This work**

NA – not available; TS –tensile strength; EB - elongation at break; HSs – hard segments; SSs – soft segments; PS/PA-amide – polystyrene endcapped with polyacrylate amide; PHEMA - poly(2-hydroxyethyl methacrylate); SHWPU - self-healing waterborne polyurethane; PTMG-1000 - poly(tetramethylene glycol) 1000; TPU - thermoplastic polyurethane; PUPCL - polycaprolactone (PCL)-diol based polyurethanes (PUs); PUU - poly(urea-urethane); PPGTD - poly(propylene glycol), tolylene 2,4-diisocyanate terminated; LDI - lysine diisocyanate; PUU–g-C_3N_4NS - poly(urea-urethane)–graphitic carbon nitride nanosheet; WPUs - waterborne polyurethanes; PEG-1450 - polyethylene glycol 1450; PU – polyurethane; PU-PEGMA – polyurethane (PU)-poly(ethylene glycol) methacrylate(PEGMA); HDI - hexamethylene diisocyanate; 2,4-TDI - toluene 2,4-diisocyanate; IPDI - isophorone diisocyanate; PCL – polycaprolactone; HEMA - 2-hydroxyethyl methacrylate.

Supplementary Table 4. Summary of the stretchable and healable triboelectric nanogenerators.

Work	Polymer Matrix	Current collector	Stretchability (%)	Healability	Performance		Ref.
					Voltage	Current	
1	PDMS	Ag NW	100	NS	50V	6 μA	[12]
2	PU	AgNW/PEDOT:PSS/PU	50	NS	70V	1.5 mAm^{-2}	[13]
3	VHB Tape	PVA based ionic conductor	700	Healable	60 V	6 μAcm^{-2}	[14]
4	VHB Tape	PAA ionic conductor	1160	NS	145 V	1.5 μA	[15]
5	PDMS	PAA−alginate ionic conductor	500	NS	100 V	0.36 μA	[16]
6	PDMS	PAA based ionic conductor	330	NS	252 V	20 μA	[17]
7	PDMS	Ag NW/PEDOT Film	50	Healable	100 V,	1 μA	[18]
8	PUA	PUA + liquid metal + Ag flakes	2500	Healable	110	4 μAcm^{-2}	This work

NS - not self-healing, PDMS - polydimethylsiloxane, PAA - polyacrylamide, PEDOT - poly(3,4-ethylenedioxythiophene), Ag - silver, NW - nanowire.

Supplementary Note 17.

The obtained voltage output and current density of our SH-TENG is one of the best reported values for deformable TENG. In terms of stretchability, the obtained stretchability of 2500 % is the highest and more than double compared to the best reported values.

Supplementary description of movies

Supplementary movies 1. The PUA sample is stretched to demonstrate the superior mechanical properties.

Supplementary movies 2. The PUA sample is stretched before mechanical damage and after healing.

Supplementary movies 3. The conductor (PUA+Ag+lq metal) sample is stretched to demonstrate the superior mechanical properties.

Supplementary movies 4. Voltage output of the SH-TENG (by finger tapping) before mechanical damaged and after healing.

Supplementary movies 5. Powering of LEDs by SH-TENG. 20 LEDs were powered by tapping the SH-TENG with finger at 0 % axial strain and by tapping the SH-TENG with palm at 2500 % axial strain, thus demonstrating its ability to act as a power source for deformable electronics.

Supplementary References

1. Narine, S. S., Kong, X., Bouzidi, L. & Sporns, P. Physical properties of polyurethanes produced from polyols from seed oils: i. elastomers. *J. Am. Oil Chem. Soc.* **84,** 55–63 (2006).
2. Chen, Y., Kushner, A. M., Williams, G. A. & Guan, Z. Multiphase design of autonomic self-healing thermoplastic elastomers. *Nat Chem* **4,** 467–472 (2012).
3. Guo, K. *et al.* Conductive elastomers with autonomic self-healing properties. *Angew. Chemie Int. Ed.* **54,** 12127–12133
4. Xiao, Y., Huang, H. & Peng, X. Synthesis of self-healing waterborne polyurethanes containing sulphonate groups. *RSC Adv.* **7,** 20093–20100 (2017).
5. Kim, S.-M. *et al.* Superior toughness and fast self-healing at room temperature engineered by transparent elastomers. *Adv. Mater.* **30,** 1705145 (2018)
6. Ur Rehman, H. *et al.* Self-healing shape memory pupcl copolymer with high cycle life. *Adv. Funct. Mater.* **28,** 1704109
7. Xu, J. H., Ye, S., Ding, C. Di, Tan, L. H. & Fu, J. J. Autonomous self-healing supramolecular elastomer reinforced and toughened by graphitic carbon nitride nanosheets tailored for smart anticorrosion coating applications. *J. Mater. Chem. A* **6,** 5887–5898 (2018).
8. Zhang, F. *et al.* A biomimetic hierarchical structure with a hydrophilic surface and a hydrophobic subsurface constructed from waterborne polyurethanes containing a self-assembling peptide

extender. *J. Mater. Chem. B* **6,** 4326–4337 (2018).

9. Ji, S., Cao, W., Yu, Y. & Xu, H. Visible-light-induced self-healing diselenide-containing polyurethane elastomer. *Adv. Mater.* **27,** 7740–7745 (2015).
10. Lin, Y. & Li, G. An intermolecular quadruple hydrogen-bonding strategy to fabricate self-healing and highly deformable polyurethane hydrogels. *J. Mater. Chem. B* **2,** 6878–6885 (2014).
11. Chen, W. *et al.* Shape-memory and self-healing polyurethanes based on cyclic poly(ε-caprolactone). *Polym. Chem.* **7,** 6789–6797 (2016).
12. Fang, H. *et al.* A stretchable nanogenerator with electric/light dual-mode energy conversion. *Adv. Energy Mater.* **6,** 1600829 (2016).
13. Hwang, B.-U. *et al.* Transparent stretchable self-powered patchable sensor platform with ultrasensitive recognition of human activities. *ACS Nano* **9,** 8801–8810 (2015).
14. Kaushik, P. *et al.* Highly transparent, stretchable, and self-healing ionic-skin triboelectric nanogenerators for energy harvesting and touch applications. *Adv. Mater.* **29,** 1702181
15. Pu, X. *et al.* Ultrastretchable, transparent triboelectric nanogenerator as electronic skin for biomechanical energy harvesting and tactile sensing. *Sci. Adv.* **3,** e1700015 (2017).
16. Liu, T. *et al.* Triboelectric-nanogenerator-based soft energy-harvesting skin enabled by toughly bonded elastomer/hydrogel hybrids. *ACS Nano* **12,** 2818–2826 (2018).
17. Lee, Y., Cha, S. H., Kim, Y.-W., Choi, D. & Sun, J.-Y. Transparent and attachable ionic communicators based on self-cleanable triboelectric nanogenerators. *Nat. Commun.* **9,** 1804 (2018).
18. Sun, J. *et al.* Self-Healable, Stretchable, Transparent Triboelectric Nanogenerators as Soft Power Sources. *ACS Nano* **12,** 6147–6155 (2018).
