# Supplementary material for: Extremely stretchable and self-healing conductor based on thermoplastic elastomer for all-three-dimensional printed triboelectric nanogenerator
Source: Nat Commun. 2019 May 14;10:2158. doi: 10.1038/s41467-019-10061-y (PMC6517406; doi:10.1038/s41467-019-10061-y)
Supplement: Supplementary file 2 — Description of Additional Supplementary Files [file 41467_2019_10061_MOESM2_ESM.pdf]

Supplementary description of movies

Supplementary movies 1. The PUA sample is stretched to demonstrate the superior mechanical properties.

Supplementary movies 2. The PUA sample is stretched before mechanical damage and after healing.

Supplementary movies 3. The conductor (PUA+Ag+lq metal) sample is stretched to demonstrate the superior mechanical properties.

Supplementary movies 4. Voltage output of the SH-TENG (by finger tapping) before mechanical damaged and after healing.

Supplementary movies 5. Powering of LEDs by SH-TENG. 20 LEDs were powered by tapping the SH-TENG with finger at 0 % axial strain and by tapping the SH-TENG with palm at 2500 % axial strain, thus demonstrating its ability to act as a power source for deformable electronics.
